# Transcriptome dynamics during metamorphosis of imaginal discs into wings and thoracic dorsum in *Apis mellifera* castes

**DOI:** 10.1186/s12864-021-08040-z

**Published:** 2021-10-22

**Authors:** Michelle Prioli Miranda Soares, Daniel Guariz Pinheiro, Flávia Cristina de Paula Freitas, Zilá Luz Paulino Simões, Márcia Maria Gentile Bitondi

**Affiliations:** 1 Departamento de Genética, Faculdade de Medicina de Ribeirão Preto, Universidade de São Paulo, Av. Bandeirantes 3900, 14049-900 Ribeirão Preto, SP Brazil; 2 Departamento de Tecnologia, Faculdade de Ciências Agrárias e Veterinárias, Universidade Estadual Paulista Júlio de Mesquita Filho, Jaboticabal, SP Brazil; 3 Instituto Carlos Chagas, FIOCRUZ, Curitiba, PR Brazil; 4 Departamento de Biologia, Faculdade de Filosofia, Ciências e Letras de Ribeirão Preto, Universidade de São Paulo, Av. Bandeirantes 3900, 14040-901 Ribeirão Preto, SP Brazil

**Keywords:** *Apis mellifera*, Honeybee, Wing imaginal discs, Metamorphosis, Caste differential expression, RNA-seq, miRNA

## Abstract

**Background:**

Much of the complex anatomy of a holometabolous insect is built from disc-shaped epithelial structures found inside the larva, i.e., the imaginal discs, which undergo a rapid differentiation during metamorphosis. Imaginal discs-derived structures, like wings, are built through the action of genes under precise regulation.

**Results:**

We analyzed 30 honeybee transcriptomes in the search for the gene expression needed for wings and thoracic dorsum construction from the larval wing discs primordia. Analyses were carried out before, during, and after the metamorphic molt and using worker and queen castes. Our RNA-seq libraries revealed 13,202 genes, representing 86.2% of the honeybee annotated genes. Gene Ontology analysis revealed functional terms that were caste-specific or shared by workers and queens. Genes expressed in wing discs and descendant structures showed differential expression profiles dynamics in premetamorphic, metamorphic and postmetamorphic developmental phases, and also between castes. At the metamorphic molt, when ecdysteroids peak, the wing buds of workers showed maximal gene upregulation comparatively to queens, thus underscoring differences in gene expression between castes at the height of the larval-pupal transition. Analysis of small RNA libraries of wing buds allowed us to build miRNA-mRNA interaction networks to predict the regulation of genes expressed during wing discs development.

**Conclusion:**

Together, these data reveal gene expression dynamics leading to wings and thoracic dorsum formation from the wing discs, besides highlighting caste-specific differences during wing discs metamorphosis.

**Supplementary Information:**

The online version contains supplementary material available at 10.1186/s12864-021-08040-z.

## Background

**Fig. 1:**
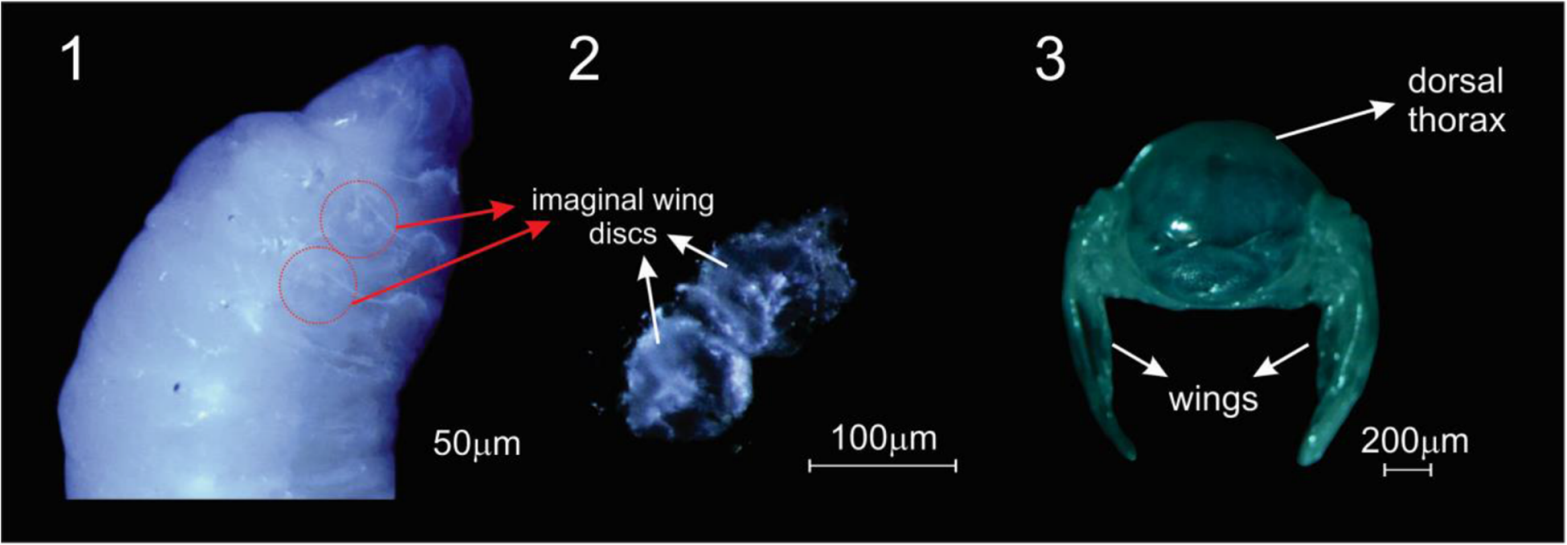
Localization of imaginal wing discs in larvae and their descendants in pupae. (1) Imaginal wing discs localization in fifth instar feeding larvae (L5F1). (2) Imaginal wing discs as dissected from an L5F1 larvae. (3) The imaginal wing discs descendants, wings and thoracic dorsum, dissected from a newly-ecdysed pupae (PW phase)

Precursors of adult structures, the imaginal discs are found in specific locations inside the larva of holometabolous insects. Imaginal discs are inward folded epithelial structures formed from local thickenings of the epidermis, the discs proper, and a squamous peripodial epithelium. Most discs also contain mesodermal myoblasts, also named adepithelial cells. During metamorphosis, whereas most of the larval tissues undergo programmed cell death, the pairs of lateral imaginal discs turn into external structures like antennae, legs, and wings; a single medial posterior imaginal disc originates the genitals [1]. It should be noted that the wing discs contribute both to the wings and notum [2], the dorsal portion of the insect thorax [3] (Fig. 1).

The imaginal discs do not differentiate until the late larval stage when they undergo a complex process of remodeling to form the adult structures during the metamorphic molt that transforms the larva into pupa [4]. The larval-to-pupal transition is coordinated by ecdysone [5, 6], which is produced from cholesterol in the prothoracic glands and is modified into its active form, 20-hydroxyecdysone (20E), in peripheral tissues [7]. The classic Ashburner model led to the knowledge of how ecdysone triggers a cascade of gene expression during metamorphosis. Briefly, by binding to a heterodimeric protein receptor, EcR-Usp, ecdysone activates the transcription of primary response genes, which ultimately activates the expression of secondary response genes that function in the successive steps leading to larval-to-pupal transition [8]. Ecdysone is necessary for the growth of imaginal tissues in last instar *Drosophila* larvae, which apparently occurs via the gene *Thor*, also known as *4E-BP* [9]. This is mediated by the insulin/insulin-like growth factor (IIS) and Target of Rapamycin (TOR) pathways [10]. Ecdysone is also necessary for imaginal disc elongation and eversion, both events being fundamental for appendage morphogenesis [4].

In contrast, the other morphogenetic hormone of insects, the juvenile hormone (JH), prevents the 20E-induced growth and differentiation of wing discs. At larval molts, JH prevents imaginal discs from initiating morphogenesis early by modulating ecdysteroids action. Ablation of the corpora allata, the pair of glands producing JH, in *Manduca sexta,* causes wing discs growth. Another component regulating wing discs metamorphosis is nutrition. Starvation impairs wing disc growth in the presence of JH but not in its absence [11]. These and other findings indicate that insulin is the nutrient-dependent signal interacting with JH and 20E for wing disc metamorphosis [12].

Among social insects, the honeybee has served as a model organism for studies on differential morphogenesis and gene expression leading to the worker and queen morphotypes [13, 14]. The reproductive queen and the optionally sterile workers living in the colony both develop from fertilized, diploid eggs. Whether the egg will give rise to a queen or a worker will depend on nutrition. The permanent nutrition with royal jelly, a mixture of secretions from the hypopharyngeal and mandibular glands produced by young worker bees, drives queen development. It has long been believed that, as the prospective queens, the prospective worker larvae receive royal jelly, but only up to day 3 after hatching. Then this diet is changed to a worker jelly, supplemented with pollen and honey [15]. However, differences in sugar content and chemical and mineral compositions have been found in the foods received by prospective queens and workers as early as on the first and second day after hatching [16, 17]. Therefore, how early prospective queen and worker larvae are fed different diets is still an open question.

In the honeybee, developmental timing from the first larval instar to the feeding phase of the fifth larval instar is the same for the worker- and queen-destined castes. But the rate of development begins to accelerate in queen larvae from the stage when they stop feeding (spinning larvae) in preparation for the metamorphic molt [18]. A subsequent apolysis event characterized by the progressive detachment of the last larval cuticle initiates the metamorphic molt [19] and is triggered by an ecdysteroid pulse [20]. Apolysis progresses from the anterior to the posterior tip of the larval body and is accompanied by the synthesis of the pupal cuticle. During this period, the developing pupa is still covered by the cuticle of the last larval instar, and thus it is named pharate-pupa. The completely formed pupa is then released from the larval cuticle during the pupal ecdysis event. As a consequence of the accelerated development, the transition to the pupal stage starts earlier in queens [18]. The last apolysis event [21], also triggered by an ecdysteroid pulse [22, 23], induces the detachment of the pupal cuticle and onset of adult cuticle synthesis. Thenceforward, the adult development occurs inside the pupal cuticle, thus characterizing the sequential pharate-adult phases. Finally, the adult insect is released from the pupal cuticle (adult ecdysis) and emerges (eclodes) from the brood cell. Ecdysis to the adult stage occurs earlier in queens in comparison to workers. Therefore, queen and worker development from newly-laid eggs to imagoes lasts about 15–16 days and 19.8–20.3 days, respectively, for the Africanized hybrids used in the current study [24].

From studies in *Drosophila*, it is known that the wing disc cells proliferate when larvae feed avidly and grow up. As larvae stop feeding, the wing discs cease growth and cell division in preparation for the subsequent ecdysteroid-induced metamorphic events. The wing discs each comprise two well-defined domains. One of them will give rise to the proximal hinge and distal wing blade, and the other will originate the notum. Sequentially, the morphogenetic events taking place in the wing discs are wing disc eversion, elongation, flattening of the wing pouch to form wing blade, accumulation of fluid between the dorsal and ventral epithelial wing disc layers, secretion of a cuticle, apposition of both epithelial layers, wing hinge contraction, vein formation, wing expansion and folding, pigmentation and sclerotization of the wings and notum. After the adult ecdysis, a final event is the spreading of the wings through filling their veins with fluid [25].

Molecular studies have provided the framework regarding signals and genes involved in the specification and patterning of the wing discs. How gene expression is interpreted by cells and tissues to pattern the body and appendages is a research area that has experienced a great progress but of which we still know little. Most of the knowledge on genetics and morphogenetic events related to wing shaping from the imaginal discs has been, undoubtedly, obtained from *Drosophila* [25, 26]. Due to the interest in the evolutionary origin of the insect wing, molecular studies on wing gene expression in basal insects [27] and other insects, such as *Tribolium castaneum* [28], *Tenebrio molitor* [29], and *Blattella germanica* [30] are critical for approaches from an evo-devo perspective [31]. Additional contributions to this important biological issue certainly are in-depth analyses of the transcriptome of imaginal discs throughout their development. This was done for wing discs of *Bombyx mori* [32, 33]. However, comprehensive comparative studies will only be possible as more transcriptomes are profiled from developing wing discs of different insects. In this direction, using RNA-seq analysis, we aimed to explore the changes in gene expression required for the morphogenetic events transforming the wing imaginal discs into adult wings and notum in the honeybee, *Apis mellifera*. This analysis was expanded to comprise the honeybee caste dimorphism that becomes particularly evident prior to the metamorphic molt, in the last larval instar, when the developmental rate is accelerated in queens [18]. In addition to differences in growth rate and body size, caste morphology dimorphism becomes progressively evident as internally, in the ovaries, for example [34], as externally, in the head and abdomen [3], and wings [35]. Characterization of genes involved in this dimorphism is essential for further reconstruction of gene regulatory networks leading to the development and differentiation of such complex morphological traits. The genetic basis of insect polyphenisms has been studied in a few species and using different approaches, as examples, absence of wings in ant workers [36], reduction of ovaries in honeybee workers [34], horns polyphenism in beetles [37], and soldier morphogenesis in a termite [38]. As a contribution, our data on transcriptome profiles of wing discs and descendant structures of workers and queens shed light on the genes expressed before the metamorphic molt in the 5th instar feeding larvae (L5F1 phase), at the height of the metamorphic molt in pharate pupae (L5PP2 phase), and after the metamorphic molt in pupae (PW phase), early (PB phase) and late (PBD phase) pharate adults. These five developmental points represent critical steps of undifferentiated imaginal wing discs transition to the pigmented and hardly sclerotized adult wings/thoracic dorsum (external morphology and characteristics of these developmental phases are shown in Additional file 1: Supplementary Figure 1 and Additional file 2: Supplementary Table 1). In addition, we conducted a computational analysis of small RNA libraries obtained from wing buds at the peak of the metamorphic process. Mature miRNAs were identified and annotated. Their regulatory roles were investigated by searching for binding sites in the 3’UTR of the mRNAs differentially expressed at the same stage of metamorphosis. Interaction networks were reconstructed to highlight mRNAs targeted by miRNAs potentially taking part in post-transcriptional gene regulation, thus providing new insights into the genetic background of wing discs metamorphosis.

## Results

### RNA-seq data set quality

The total number of genes identified in the 30 RNA-seq libraries obtained from the wing imaginal discs and their derived body parts, i.e., wings and thoracic dorsum (Fig. 1), of workers and queens was 13,202. This number corresponds to 86.2% of the 15,314 genes annotated for the honeybee genome (Custom honeybee putative ortholog database at the Amel_4.5 GenBank assembly accession: GCA_000002195.1).

To assess the variation in gene expression in the RNA-seq data sets, we determined the coefficient of variance (CV^2^) for each group of three independent biological samples (triplicate) tested for each of the five developmental phases of workers and queens (see samples preparation in Methods section). As expected, the variance was higher for the less expressed genes, i.e., when the average expression (FPKM) was low (Additional file 3: Supplementary Figure 2). We also determined the expression level dispersion patterns for the triplicates. A higher variability (large dispersion) was evident for the less expressed transcripts than for the more expressed transcripts, which showed small dispersion (Additional file 4: Supplementary Figure 3).

### Cluster analyses grouped the transcriptomes according to the phase of wing discs differentiation to adult wings and thoracic dorsum, independent of the caste

**Fig. 2:**
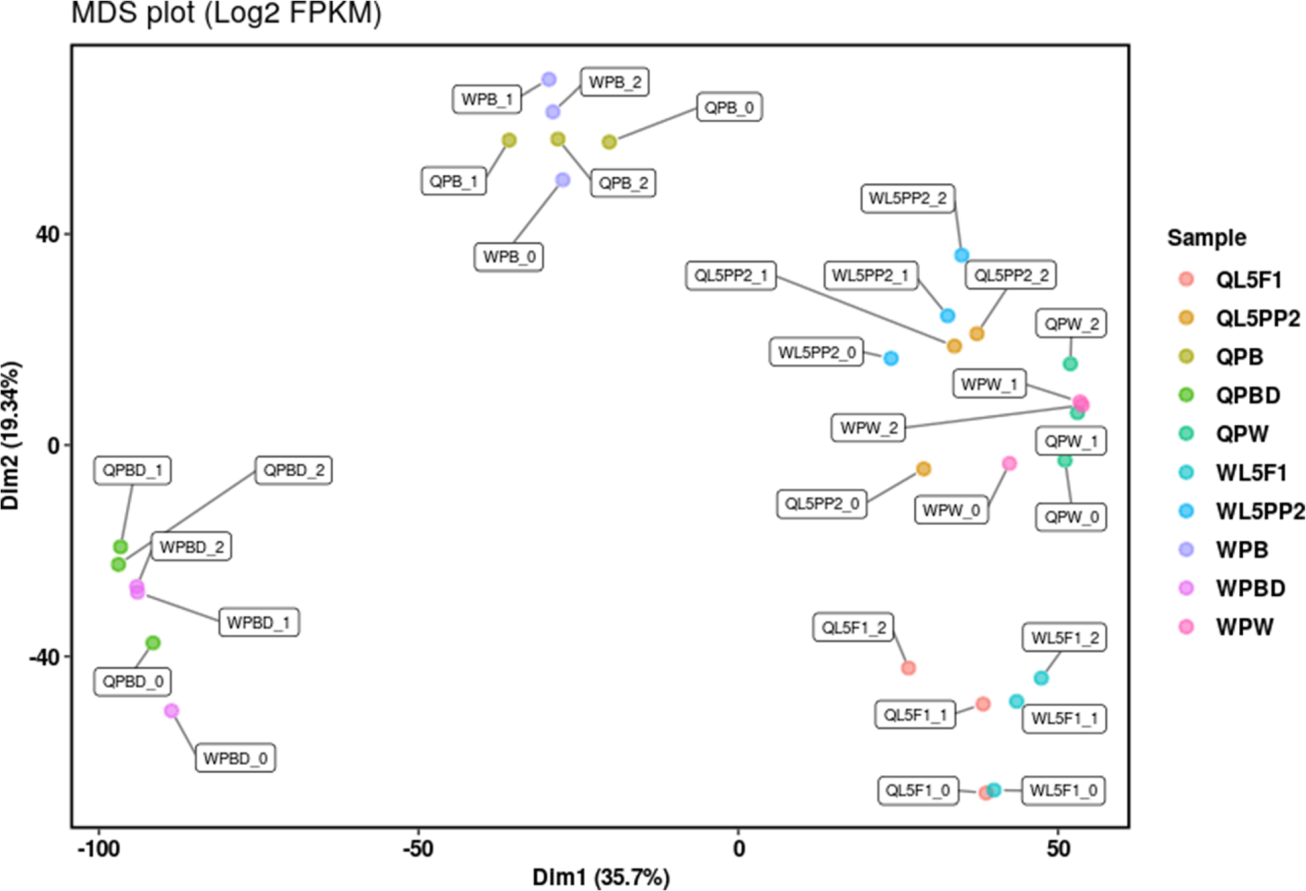
Multidimensional scaling (MDS) plot showing the relationships (similarity/dissimilarity) between the RNA-seq libraries of wing imaginal discs and their developing descendants, i.e., thoracic dorsum and wings. Worker (W) and queen (Q) samples are indicated for each developmental phase (at the right) from which we extracted the imaginal discs and descendants. L5F1 phase (undifferentiated imaginal discs), L5PP2 phase (metamorphosing imaginal discs), PW phase (pupal wing/thoracic dorsum), PB phase (immature adult wing/thoracic dorsum), PBD phase (mature adult wing/thoracic dorsum). Independent biological samples of a same developmental phase are indicated by _0, _1, and _2

Differences and similarities among wing discs and descendant structures from workers and queens were analyzed through multidimensional scaling using a similarity matrix to plot a graph representing the distances between the RNA-seq libraries (Fig. 2). This analysis, including all the 13,202 expressed genes, grouped worker and queen samples according to the developmental phases. Thus, premetamorphic wing disc libraries from workers and queens at the L5F1 phase clustered together; two other clusters were formed by wings/thoracic dorsum libraries from workers and queens at the early (PB phase) and late (PBD phase) pharate-adult phases. A fourth cluster grouped the libraries of the successive L5PP2 and PW phases representing wing discs in metamorphosis and pupal wings/thoracic dorsum, respectively.

**Fig. 3:**
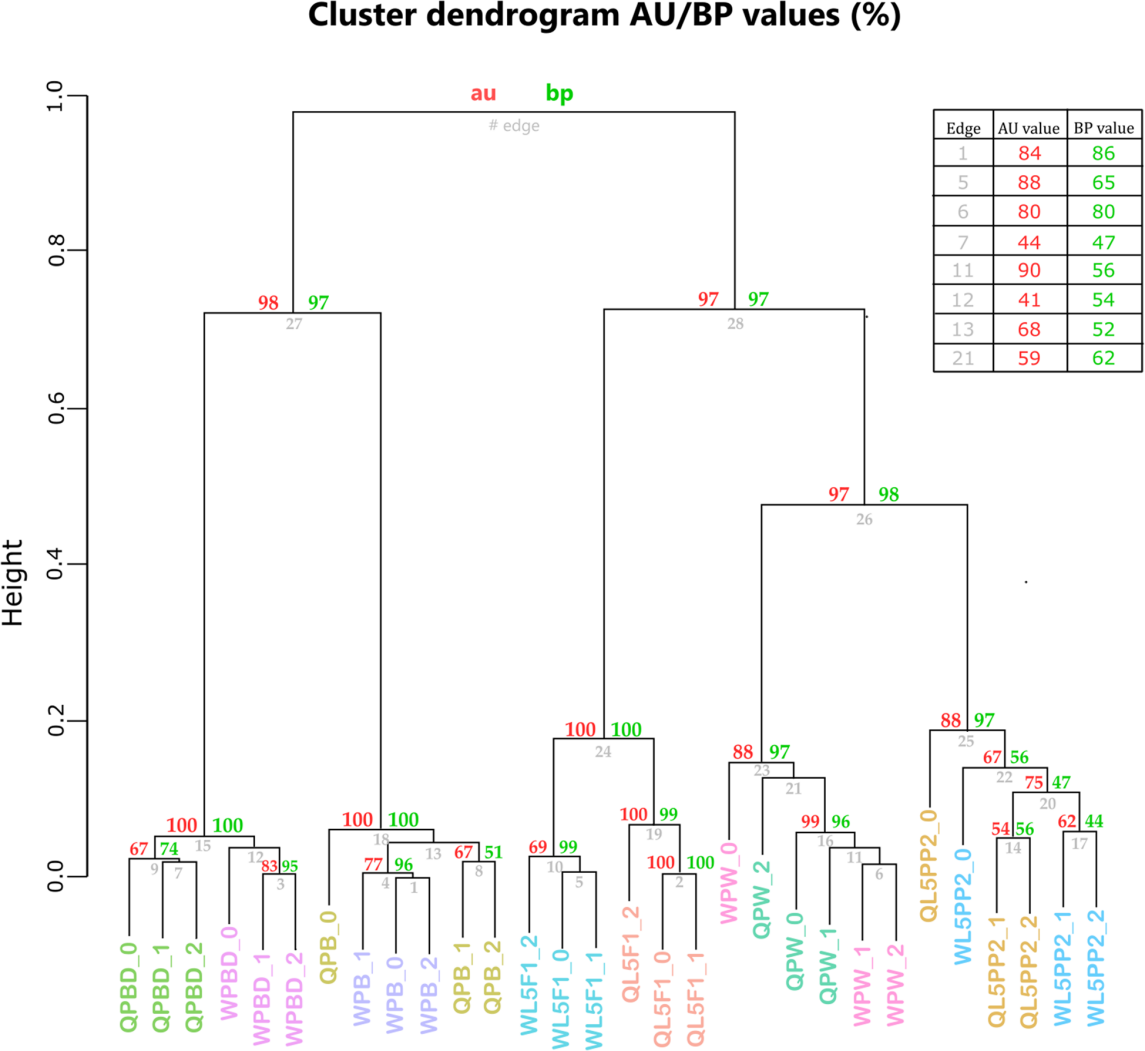
Dendrogram representing the Euclidean distance analysis grouping the DEGs according the developmental phase (L5F1, L5PP2, PW, PB, or PBD). The letters W and Q ahead of each developmental phase acronym represent workers and queens, respectively. Independent biological samples of a same developmental phase are indicated by _0, _1, and _2. AU cluster supporting values are in red. Branch BP supporting values (10,000 replications) are in green. Edge values are in grey. The small table on the right shows the AU and BP values (corresponding to edges 1, 5, 6, 7, 11, 12, 13, and 21) that could not be indicated in the dendrogram due to insufficient space

A similar result was obtained when we applied Euclidean distance analysis to the differentially expressed genes (DEGs) set. A False Discovery Rate of 0.05 for identification of false positives was established for the statistical analysis of differential gene expression. Aiming to reduce false positives, we also adopted the adjusted *p*-value (q-value) < 0.05, log_2_ fold-change ≥1 and ≤ − 1, and FPKM ≥5. The Euclidean distance analysis separated the DEGs into five distinct clusters representing the phases of imaginal discs development to wings/thoracic dorsum. Note that workers and queens at the same phase were grouped together. These clusters are supported by high values of AU and BP (Fig. 3). Consistency was also found at a higher level of the dendrogram, where we could observe the clustering of samples at the metamorphic phase (L5PP2) and newly-ecdysed pupal phase (PW) (AU = 97; BP = 98) (Fig. 3) as evidenced by the multidimensional scaling.

### Gene expression through the phases of wing discs development and between castes, and functional analysis

**Fig. 4:**
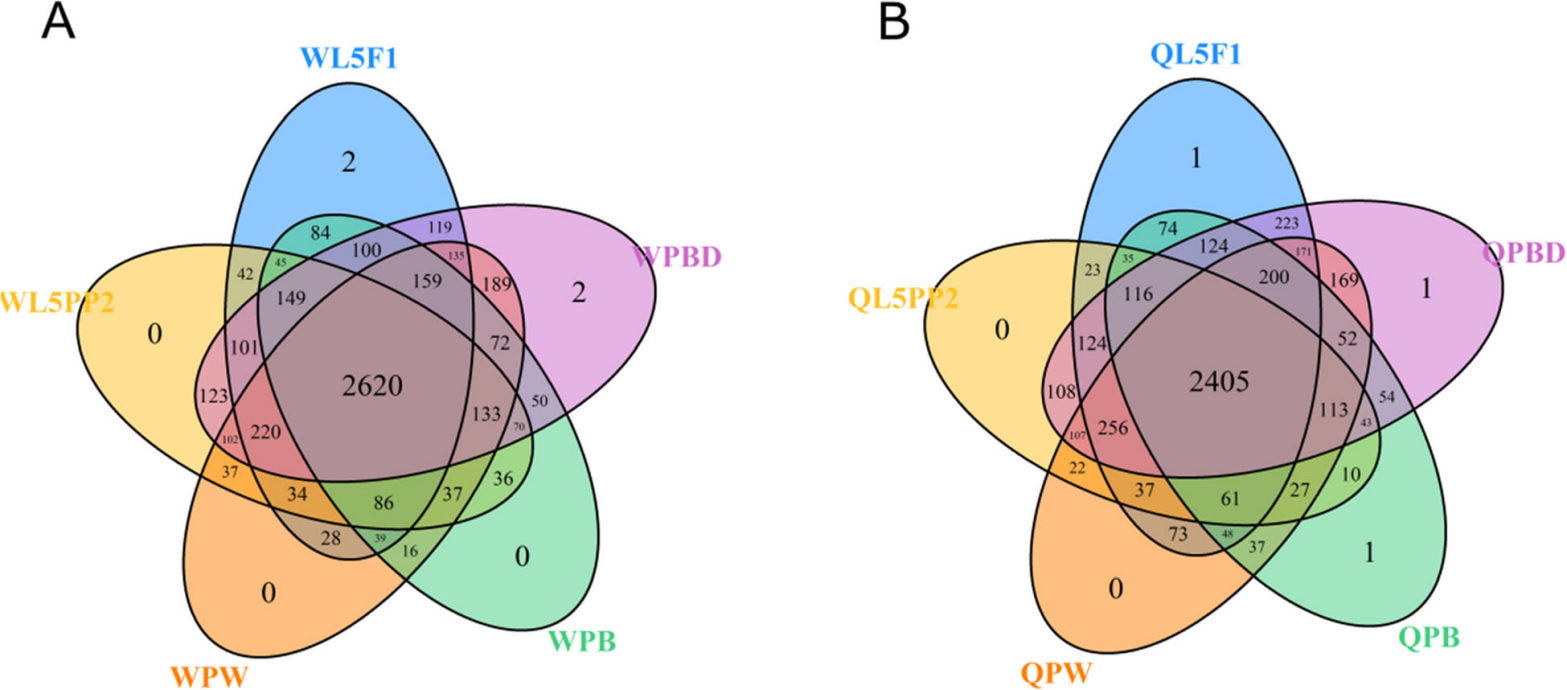
Number of DEGs co-expressed or exclusively expressed in wing discs and their descendants through developmental phases of (**A**) workers and (**B**) queens

Venn graphs were constructed and highlighted the number of genes shared by, or exclusive of, the developmental phases of workers (Fig. 4a) and queens (Fig. 4b) encompassing wing discs development to wings and thoracic dorsum. From the 4830 DEGs detected in workers, 2620 were expressed in all developmental phases. Transcripts for two genes (LOC100577577 and LOC102654781) were specific to premetamorphic wing discs (L5F1 phase), and for other two genes (LOC408608 and LOC725202) were specific to the late pharate-adult wings/thoracic dorsum (PBD phase). For queens, 2405 genes from the 4715 DEGs were expressed in all developmental phases. Developmental phase-specific transcripts were found in the premetamorphic wing discs of the L5F1 phase (LOC102654781), and in the wings/thoracic dorsum of the early pharate-adult PB phase (LOC102654781) and late pharate-adult PBD phase (LOC102655007). It was possible to attribute Gene Ontology (GO) function only to one of these exclusively expressed genes. The gene identified as LOC100577577 in workers at the L5F1 phase has function related to chitin-binding and chitin metabolic process (http://hymenopteragenome.org/hymenopteramine/begin.do).

**Fig. 5:**
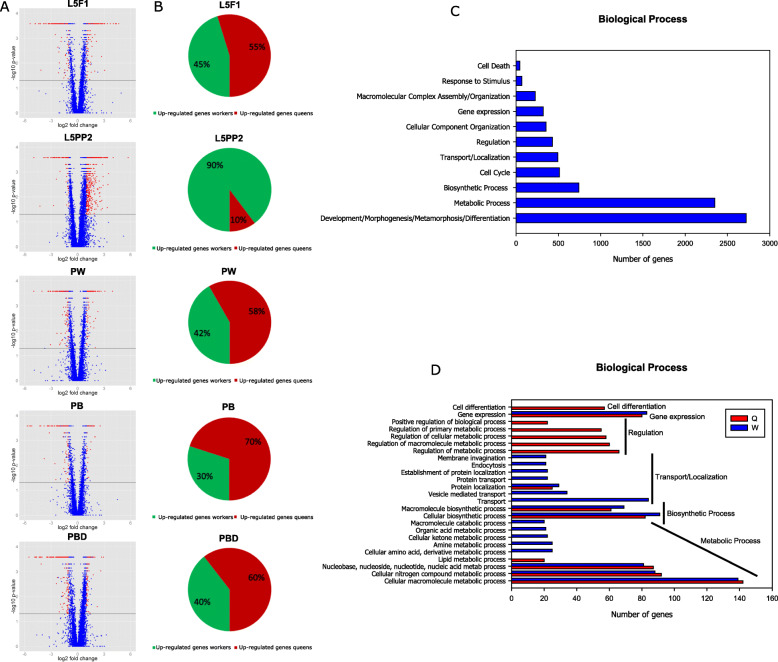
Differential gene expression during imaginal disc development to wings/thoracic dorsum in workers and queens. **A** Volcano graphs represent the upregulated and downregulated DEGs (red dots at the right and left of the volcano graphs, respectively) among the total of genes (blue plus red dots) identified in the RNA-seq analysis. The horizontal line in each volcano graph indicates the adjusted *p*-value (0.05). **B** Percentage of upregulated genes in workers or queens. (C) Gene Ontology (GO) functional analysis of DEGs displaying similar expression in workers and queens (see Additional file 5: Supplementary Table 2). (D) GO analysis of DEGs upregulated in the developing wing discs of workers or queens (see Additional file 6: Supplementary Table 3)

Volcano graphs (Fig. 5a) separate for each developmental phase, the upregulated and downregulated DEGs, in the total of genes identified in the RNA-seq analysis of wing discs and their descendants in workers and queens. It was clear that in the L5PP2 phase, when the imaginal discs undergo metamorphosis, a greater number of DEGs were upregulated in comparison with the L5F1 phase (premetamorphic wing discs), PW phase (pupal wings/thoracic dorsum), PB and PBD phases (early and late pharate-adult wings/thoracic dorsum). We also show the proportion of DEGs that were upregulated in each caste (Fig. 5b). The proportion of upregulated DEGs in queens in each developmental phase was higher than in workers, except for the L5PP2 phase, where a greater proportion of upregulated genes was observed in workers than queens.

Using g:Profiler (g:Orth) and DAVID tools, we searched for the honeybee DEGs displaying orthology relationships with *Drosophila* genes. Those corresponding to FlyBase gene IDs (FBgn numbers) were then used for GO functional annotation (level 3). The GO enrichment analysis was done for “Biological Process” category, here considered the most informative category. For this GO analysis we used the ortholog DEGs similarly expressed in workers and queens in each developmental phase (in spite of showing differential expression through the developmental course) and also the DEGs upregulated in workers or queens within each developmental phase. Figures 5c, d represent the GO analyses for the DEGs similarly expressed in both castes and DEGs upregulated in workers or queens. We only considered for these analyses those GO terms statistically significant and including at least 20 genes.

The most representative Biological Process terms shared by both castes (Fig. 5c) could be categorized as “Development/Morphogenesis/Metamorphosis/Differentiation”, “Metabolic process”, “Biosynthetic process”, “Cell cycle”, “Transport/Localization”, “Regulation”, “Cellular component organization”, “Gene expression”, “Macromolecular complex assembly/Organization”, “Response to stimulus”, and “Cell death”. Note that the GO terms linked to “Development/Morphogenesis/Metamorphosis/Differentiation” were the most enriched in our GO analysis (Additional file 5: Supplementary Table 2). When the GO analysis was performed with the sets of DEGs upregulated in workers or queens (Fig. 5d), we observed that several of these genes shared a same “Biological Process” subcategory. Therefore, functional terms included in the “Metabolic process”, “Biosynthetic process”, “Transport/Localization”, and “Gene expression” subcategories were shared by genes upregulated in queens or workers. However, almost all the enriched GO terms related to “Transport/Localization” subcategory were worker-caste exclusive, and all the GO terms related to “Regulation” and “Differentiation” showed to be queen-caste exclusive (Additional file 6: Supplementary Table 3).

We also searched for significantly enriched pathways between and within the two honeybee castes. The 20 most relevant pathways shared by both castes, or enriched in queens or workers are listed in Additional file 7: Supplementary Table 4. The 20 shared pathways could be included in the following higher hierarchical levels: “Metabolism of Proteins”, “Metabolism of RNA”, “Metabolism”, and “Response to Stimuli”. For queens, the 20 most significant pathways were linked to the following main categories “Metabolism of RNA”, “DNA repair”, “Gene expression (transcription)”, “Metabolism of Proteins”, and “Protein Localization”. Workers showed a greater diversity of pathways among the 20 most significant ones: “Metabolism of Proteins”, “Metabolism of RNA”, “Cellular Response to Stimuli”, “Signal Transduction”, “Immune System”, “Metabolism”, “Cell-cell Communication”, “Cell-cycle”, and “Vesicle-mediated Transport”.

### Selected DEGs represented in the transcriptomes

#### Ecdysis-related genes

**Fig. 6:**
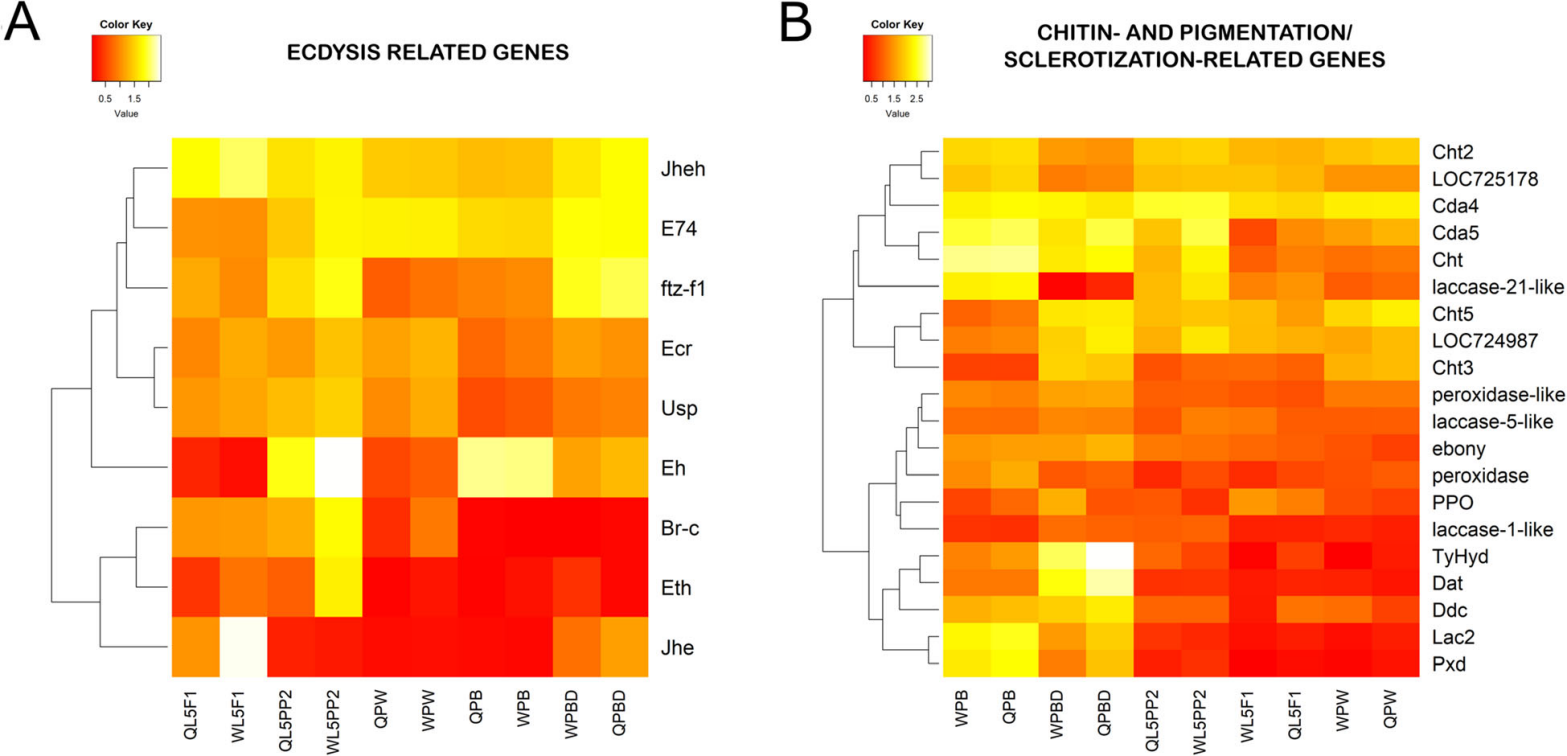
Expression profiles of (**A**) genes related to ecdysis and (**B**) genes encoding chitin- and cuticle pigmentation/sclerotization-related enzymes in the wing discs and developing wings/thoracic dorsum of workers and queens in the different developmental phase

A supervised hierarchical clustering allowed us to compare 9 ecdysis-related genes in a heatmap based on their expression profiles (Fig. 6a). These genes encode the ecdysone receptors EcR (Ecdysone Receptor) and Usp (Ultraspiracle), and Br-c (Broad complex), E74, and Ftz-f1 (Fushi tarazu-f1) transcription factors, which are all part of the cascade induced by the binding of ecdysone to the EcR/Usp complex. In this group, we also included the genes *Eh* and *Eth*, which encode Eclosion- and Ecdysis-triggering hormones, respectively, and also the genes *Jhe* and *Jheh* for the JH degrading enzymes, JH-esterase (JHE) and JH-epoxide hydrolase, that control JH titer and effect.

Transcripts for EcR and Usp did not show clear increased levels; their expression profiles clustered together, suggesting a timed action through wing disc development. In contrast, a *Br-c* transcripts peak was evident in the metamorphosing wing discs of workers in the L5PP2 phase, when the ecdysteroid titer also peaks [20], suggesting induction by the hormone. Like *Br-c*, the genes *E74*, *ftz-f1, Eh*, and *Eth* were all more expressed in the wing discs of workers than queens at the L5PP2 phase. However, *E74*, *ftz-f1*, and *Eh* have also expressed in other developmental phases and ecdysteroid levels conditions. Thus, *E74* also showed a high expression in workers and queens in conditions of low and basal ecdysteroid titers [22] at the PW and PBD phases, respectively; *ftz-f1* was reinduced in both castes at the PBD phase; and *Eh* was also reinduced in workers as well as in queens at the PB phase, when the ecdysteroid titer is decaying [22].

The heatmap also showed that *Jhe* is maximally induced in the wing discs of workers but not in the wing discs of queens at the L5F1 phase, which is consistent with a caste-specific level of JH degradation at this stage.

#### Genes involved in chitin metabolism, cuticular pigmentation, and sclerotization

We also focused on the expression profiles of genes involved in chitin metabolism and melanization/sclerotization pathway (Fig. 6b). Genes encoding chitin-related enzymes were differentially expressed during wing discs development, and sometimes, between the castes. As examples, *chitin deacetylase 5* (*Cda5*), a *chitinase* gene (*Cht*), and LOC724987 were clearly differentially expressed between castes at the L5PP2 phase. *Chitinase 5* (*Cht5*) was more expressed in queens than workers at the pupal wing/thoracic dorsum (PW phase).

Except for the *laccase-21-like* gene, which may be involved in cuticle sclerotization like verified for another member of the laccase family in the honeybee, *Lac2* [39], the melanization/sclerotization pathway genes are grouped separately from the chitin-related genes in the heatmap (Fig. 6b), thus evidencing grouping by function. In the low level of the heatmap hierarchy, the cluster of melanization/sclerotization genes subdivided into two subclusters, one of them containing genes induced in the PB/PBD pharate-adults [*tyrosine-hydroxylase* (*TyHyd*), *dopamine-N-acetyltransferase* (*Dat*), *dopa decarboxylase* (*Ddc*), *Lac2*, *peroxidase* (*Pxd*)]. In general, these genes showed caste-differential expression in the PBD pharate adults, and *TyHyd* was the most strongly induced in queens at this developmental phase. The other subcluster included melanization/sclerotization genes displaying less variable expression profiles [*peroxidase-like*, *laccase-5-like*, *ebony*, *peroxidase*, *prophenoloxidase* (*PPO*), and *laccase-1-like*]. The peroxidase genes, *Pxd, peroxidase*, and *peroxidase-like*, clustered together with the melanization/sclerotization related genes and are possibly involved in wings/thoracic dorsum cuticle pigmentation and hardening.

#### Cuticular protein genes

**Fig. 7:**
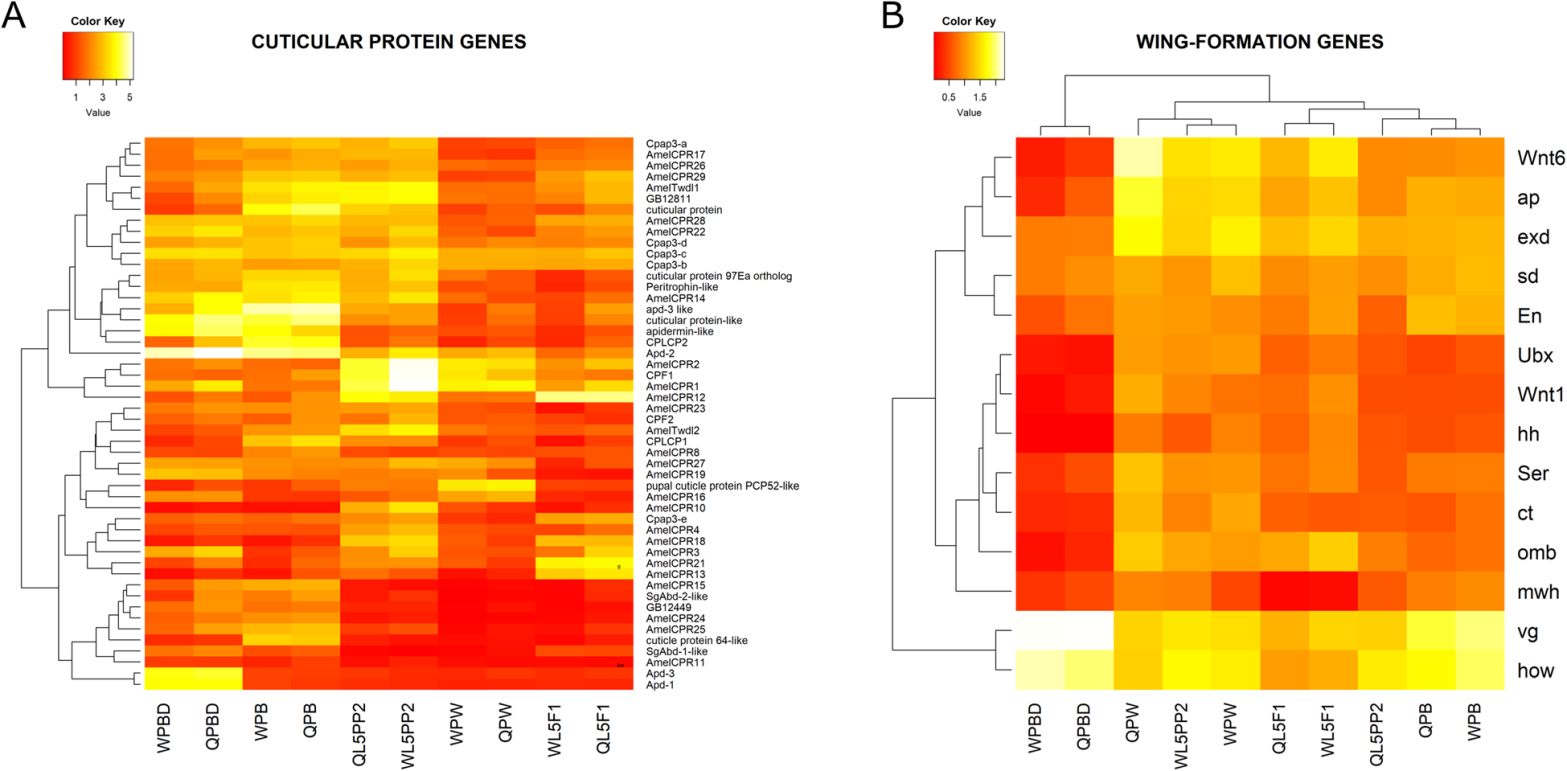
Expression profiles of (**A**) cuticular protein genes and (**B**) wing-patterning genes in workers and queens in the different developmental phases

We found 50 cuticular protein genes in our RNA-seq libraries, 41 of them encoding proteins pertaining to known cuticular protein families. Although missing in the cuticle databank (cuticleDB), the remaining 9 genes showed high similarity with other sequences of putative cuticle proteins, as verified by BLAST analysis against the NCBI databank. Figure 7a shows that the expression of these cuticle protein genes may vary during wing disc development and between the castes. The *AmelCPR21* and *AmelCPR13* genes encoding CPR family proteins were both strongly upregulated in the imaginal wing discs of the L5F1 phase. Two other CPR genes, *AmelCPR10* and *AmelCPR4*, and a Tweedle-family gene*, AmelTwdl2*, were all upregulated in the metamorphosing wing discs of the L5PP2 phase; *pupal cuticle protein PCP52-like* was upregulated in pupal wings/thoracic dorsum of the PW phase; *cuticle protein 64-like*, *CPLCP1*, *CPLCP2*, *peritrophin-like* and *cuticular protein* were all mostly upregulated in wings/thoracic dorsum of early pharate-adults (PB phase); *Apd-1* and *Apd-3* were upregulated in wings/thoracic dorsum of late pharate-adults (PBD phase). Several genes (*Apd-1*, *Apd-3*, *SgAbd1-like*, *cuticle protein 64-like*, *AmelCPR25*, *AmelCPR24*, *GB12449*, *SgAbd2-like*, and *AmelCPR15*) showed basal expression at earlier developmental phases (L5F1, L5PP2, and PW) and increased expression in pharate-adults (PB and PBD phases), which is consistent with roles in adult cuticle development triggered by the decline in ecdysteroid titer. These genes clustered together in the heatmap. In each developmental phase, we also found differential expression between the castes. As examples, *AmelCPR1*, *AmelCPR2*, and *CPF1* were strongly upregulated in the metamorphosing wing discs of workers at the L5PP2 phase in comparison to queens at the same phase; *CPLCP1* was upregulated in wings/thoracic dorsum of early pharate-adult queens (PB phase); *Apd-3 like* displayed higher expression in queens at the PBD phase. The five genes for CPAP3 family proteins in the honeybee genome were expressed in the wing discs and derivatives, without striking differences between the castes. The expression profiles of *Cpap3-b*, *Cpap3-c*, and *Cpap3-d* are more similar to each other. *Cpap3-a* and *Cpap3-e* occupy distant positions on the heatmap, evidencing differences in their expression profiles (Fig. 7a).

#### Wing patterning genes

In the wing discs and derived structures, we detected the expression of 14 genes known as involved in *Drosophila melanogaster* wing pattern formation. Orthology relationships were investigated and confirmed (Additional file 8: Supplementary Table 5). These genes showed expression levels oscillation during wing disc development (Fig. 7b). One of these genes, *vestigial* (*vg*), showed a very high expression in the wings/thoracic dorsum of the later developmental phase, PBD. Expression profiles of *vg* and *held out wings* (*how*) clustered together, suggesting a concatenated action in wing disc development. The expression patterns of *Ultrabithorax* (*Ubx*), *Wnt1* (a member of the Wnt family; *wingless* orthologous), *hedgehog* (*hh*)*, serrate (Ser)*, *cut* (*ct*), *optomotor-blind* (*omb*), *and multiple wing hair* (*mwh*) clustered together in the heatmap, separately from *vg* and *how*. During the development of the premetamorphic wing discs (L5F1 phase) to the early pharate-adult wings/thoracic dorsum (PB phase), these clustered genes showed variable expression levels with frequent differences between castes. At the end of the pharate-adult development (PBD phase) they showed basal expression levels and, virtually, no differences between castes. The other cluster in the heatmap (Fig. 7b) consists of five genes: *Wnt6* (a member of the Wnt family)*, apterous* (*ap*), *extradenticle* (*exd*), *scalloped* (*sd*)*,* and *engrailed* (*En*). Expression of *Wnt6* and *ap* is higher in queens at the PW phase and subsequently declines in queens and workers. Expression of *exd* is also high in queens at the PW phase, but it is similarly high in workers at the same phase and L5F1 phase and then decays as development progresses. Such decay is not so evident for *sd* gene expression. *En* showed maximal expression in the PB phase for queens and workers.

In summary, at the premetamorphic L5F1 phase, all the wing-forming genes, except *mwh* and *ct*, exhibited, to a greater or lesser extent, a higher expression level in workers. Similarly, during metamorphosis in the L5PP2 phase, the majority of the wing-forming genes were more expressed in workers.

### Micro RNAs

#### miRNAs expressed in the honeybee worker wing discs at the metamorphic transition

We assessed the miRNAs expressed in workers’ metamorphosing wing discs (both fore- and hindwing discs) at the L5PP2 stage. miRNA reads numbers and length distribution after quality filtering are shown in Additional file 9: Supplementary Table 6 and Additional file 10: Supplementary Figure 4, respectively. A total of 197 mature miRNAs are expressed in the metamorphosing wing discs (Additional file 11: Supplementary Table 7). Our analysis allowed the annotation of mature miRNAs originated from both arms of miRNA hairpins. We identified reads for both arms of 71 hairpins. The differences in the number of reads for each arm reinforce the existence of tissue- and/or time-specific factors that act on the arm switch mechanisms resulting in the differential accumulation of mature miRNAs. Table 1 lists ame-miRNAs whose homologs in other insect species are known as having roles in wing metamorphosis. The ame-miR-100, ame-miR-125 and ame-let-7 miRNAs are highly expressed in the wing discs and are located *in tandem* in the LG8 linkage group of the honeybee genome; members of ame-miR-2 family and ame-miR-9 are also expressed in the honeybee wing discs. Table 1 as well includes ame-miRNAs sharing homology relationships with miRNAs with described roles in larval-to-pupal transition or ecdysteroid titer control of metamorphosis. Included in Table 1 are also ame-miRNAs with other known functional activities.

**Table 1:** Honeybee miRNAs whose homologs in other insect species have been related to the regulation of metamorphosis and other developmental processes (see Discussion for references)

Function	ame-miRNAs types	ame-mir-RNAs arms
Wing metamorphosis-related	ame-miR-100	ame-miR-100-5p^b^
	ame-miR-125	ame-miR-125-5p^b^
	ame-let-7	ame-let-7-5p^b^
	ame-miR-2 family	ame-miR-2-1-3p^a^
		ame-miR-2b-5p^a^
		ame-miR-2-3-3p^a^
		ame-miR-2-2-3p^a^
		ame-miR-13b-3p^b^
		ame-miR-13a-3p^b^
	ame-miR-9	ame-miR-9a-3p^b^
		ame-miR-9b-3p^b^
Larval-to-pupal transition-related	ame-miR-31	ame-miR-31a-5p^a^
	ame-miR-275	ame-miR-275-3p^b^
	ame-miR-276	ame-miR-276-3p^b^
Ecdysteroid titer control of metamorphosis	ame-let-7	ame-let-7-5p^b^
	ame-miR-125	ame-miR-125-5p^b^
	ame-miR-100	ame-miR-100-5p^b^
	ame-miR-34	ame-miR-34-5p^b^
	ame-miR-8	ame-miR-8-3p^b^
	ame-miR-252	ame-miR-252a-5p^a^
		ame-miR-252b-5p^a^
	ame-miR-965	ame-miR-965-3p^b^
	ame-miR-bantam	ame-miR-bantam-3p^b^
	ame-miR-14	ame-miR-14-5p^b^
	ame-miR-281	ame-miR-281-3p^b^
Other reported functional activities	ame-miR-bantam	ame-miR-bantam-3p^b^
	ame-miR-8	ame-miR-8-3p^b^
	ame-miR-277	ame-miR-277-3p^a^
	ame-miR-278	me-miR-278-5p^b^
	ame-miR-252	ame-miR-252a-5p^a^
		ame-miR-252b-5p^a^

^a^ Exclusively expressed arm

^b^ More expressed arm

#### miRNA-targets in the wing discs at the metamorphic transition

**Fig. 8:**
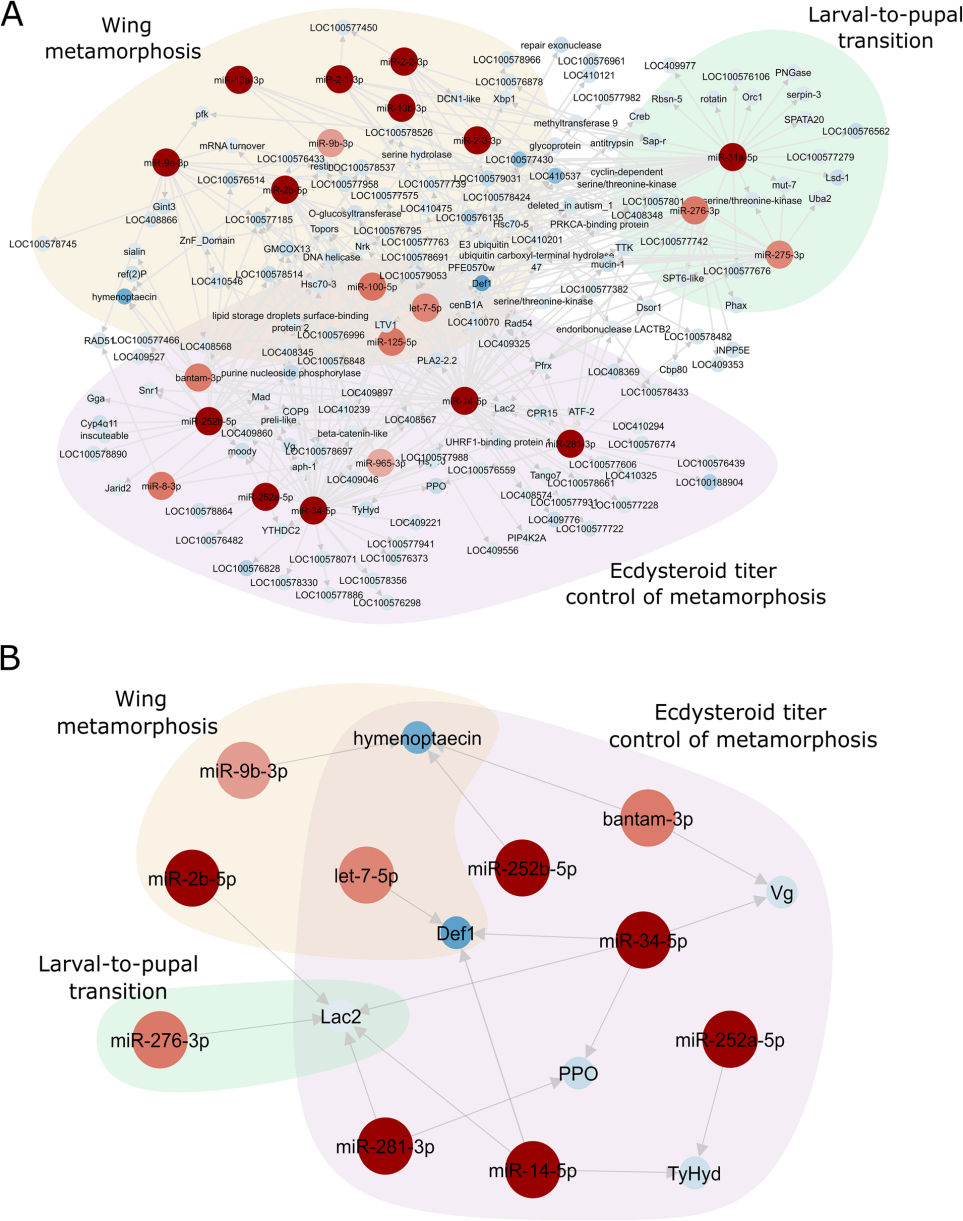
miRNAs-targets network. (**A**) Targets of 22 ame-miRNAs whose homologs in other insect species have been related to metamorphosis, larval-to-pupal transition, and ecdysteroid control of metamorphosis. (**B**) Ten ame-miRNAs among the 22 ones putatively regulating genes with described functional activities in the honeybee. The red circles represent ame-miRNAs (the darker the color, the greater the number of reads). The blue circles represent the downregulated genes in the wing discs of L5PP2 workers

To explore the regulatory roles of ame-miRNAs in wing discs metamorphosis, we predicted regulatory elements for miRNAs in the 3’UTR of DEGs in workers at the L5PP2 developmental phase. We then focused on the roles of the 22 ame-miRNAs listed in Table 1. Figure 8a represents the interaction between these ame-miRNAs and DEGs at the metamorphic peak. We could see that a significant part of the DEGs targeted by these 22 ame-miRNAs have yet non identified roles, including DEGs targeted by ame-miR-100-5p, ame-miR-125-5p, and ame-let-7-5p (Additional file 12: Supplementary Figure 5). However, 10 of these ame-miRNAs putatively regulate the levels of transcripts encoded by genes with described functional roles in the honeybee. We represented in Fig. 8b these 10 ame-miRNAs and their putative targets. Interestingly, the targets could be grouped into two classes of genes: those functionally related to the melanization/sclerotization pathway (genes *TyHyd*, *Lac2*, and *PPO*) that is critical for wing cuticle differentiation, and those with roles in the immune response, *defensin-1* (*def-1*), *hymenoptaecin* and *vitellogenin* (*Vg*).

We also searched for predictive interactions between ame-miRNAs and the 14 wing disc patterning genes (Fig. 7b). Three of these genes, *exd*, *mwh*, and *ap*, showed significant interaction with ame-miRNAs at the metamorphosis peak. The interactions are positive, suggesting that these genes are upregulated by miR-2b-5p, let-7-5p, and miR-2-3-3p, respectively (Additional file 13: Supplementary Figure 6). Although less studied, mechanisms underlying the direct or indirect gene activation by miRNAs have been proposed [40]. Functions of these three genes seem to be required at the metamorphic molt, which is consistent with the fact that we did not find miRNAs interaction with the other 11 wing patterning genes.

## Discussion

It is generally agreed that caste-specific differences are determined at the regulatory level since the genomic template of workers and queens is the same. The differential feeding of female larvae leading to the two different phenotypes affects general growth and causes differential development of body structures and organs. Key components of caste differentiation are metabolic flux, hormone titers (JH and ecdysteroids), and epigenetic regulatory mechanisms (DNA methylation, histone modifications). The phenotypic plasticity is currently being explained in terms of spatio-temporal expression of genes interacting in complex gene-regulatory networks [41, 42]. Imaginal wing discs development in workers and queens is potentially subjected to all these players that caste-specifically differentiate thorax and wings morphologies. Both honeybee castes develop functional wings (Additional file 14: Supplementary Figure 7A, B), therefore in this species the wings are not examples of extremely divergent developmental phenotypes as are the ovaries (highly developed in queens and reduced in workers) [34], and the wings in ants, which generally show winged queens and wingless workers [36]. The honeybee wing caste dimorphism is mainly expressed in the wing size (Additional file 14: Supplementary Figure 7C, D) and number of wing hooks (hamuli) [35], which are present in the anterior margin of the hindwing and serve to couple it to the posterior margin of the forewing, thus ensuring the synchronic beat of the wings during flights. Differences were also described in the number of bristles and cubital index (ratio of two of the forewing veins) [43]. Consistent with these apparently modest phenotypic differences, the developing wings of queens and workers display differential expression of several genes, which exhibit distinct GO functional classes and biological pathways. Wing shape dimorphism was also demonstrated for the forewings of three other hymenopteran species. Hornets (Vespidae), like the honeybees, show subtle morphological differences between the wings of queens and workers. In this case, there was evidence that the different wing patterns do not result from a simple scaling effect (wasp gynes are mostly larger than workers) but from changes in the developmental pathway [44].

### Transcriptomes profiles differ more between developmental phases than between castes

Distance analyses of the transcriptomes obtained from the wing imaginal discs and descendant structures revealed that gene expression displayed more variation between the developmental phases, representing the course of wing discs transformation to pupal and adult wings/thoracic dorsum, than between the female castes. These analyses clearly evidenced the clustering between the L5PP2 and PW phases, thus exposing their greater similarity in terms of gene expression landscape. In the L5PP2 stage, the pupal head and thoracic appendages are built under the larval cuticle [19], which explains the clustering of the L5PP2 and PW phases. The epithelial layer originated from the wing discs is engaged in synthesizing the pupal cuticle of the wings and thoracic dorsum. After pupal cuticle apolysis, the epidermis produces the adult cuticle, thus characterizing the PB phase. The cuticle then turns intensely pigmented and sclerotized at the PBD phase [21]. Our distance analysis also clearly separated the adult wings and thoracic dorsum from the pupal ones in terms of gene expression. In addition, the distance analyses showed that despite the differences in the development timing between workers and queens (workers take a longer time to emerge than queens) [18], the morphological criteria used for identifying correlated developmental phases [19, 24] were suitable and efficient. The independent biological samples for each developmental phase clustered together independently of the caste. The premise of preparing samples according to the developmental phase is to avoid biased gene expression due to age, thus allowing us to highlight the caste-differential gene expression at the very same stage, marked by the same developmental events. Furthermore, using different distance analyses, we obtained similar results for the set of all the identified genes as well as for the more restricted set of DEGs.

### Functional analysis revealed caste-shared and caste-specific overrepresented GO terms and biological pathways

We found that the Biological Process terms associated with “Development/Morphogenesis/Metamorphosis/Differentiation” that could intuitively be linked to wing disc development were enriched in and shared by queens and workers. We suggest that genes associated with these GO terms are markers of wing disc development independent of the caste. Similarly, GO subcategories within two other representative Biological Process terms, “Metabolic Process” and “Biosynthetic Process”, were shared by queens and workers. However, some of the “Metabolic Process” terms were caste-specific, and terms related to “Regulation” were exclusively enriched in queens, whereas terms related to Transport/Localization were enriched in workers, thus highlighting particularities in wing disc differentiation between castes. Interestingly, several of the Biological Process terms included in “Development”, “Metabolic Process”, “Regulation”, “Transport localization”, and “Cellular component organization” classes matched, or were similar, to Biological Process terms obtained from *B. mori* wing discs undergoing metamorphosis from larvae to early pupae [32]. Similarly, most GO terms found in wing discs descendants in *B. mori* older pupae were categorized as “Metabolic Process” [45], thus highlighting the importance of genes in these GO classes for wing imaginal disc development.

Among the 20 overrepresented biological pathways within and between castes, the worker wing discs and descendants showed the greater diversity of categories at the higher hierarchical level. Taken together, the differential expression of genes, GO terms, and biological pathways shown by the developing wing discs of workers and queens support the shift to alternative pathways during their respective developmental trajectories. A candidate for inducing the shift is, among others, the JH, whose titer is higher in queens comparatively to workers [20]. If so, the induction should occur before entering the metamorphic molt, when the hormone titer is lowered to basal levels in both castes, which is an essential condition for larva-pupa transition. JH is involved in general body growth and development of caste-biased structures, like the corbicula only seen in the hindlegs of workers [46]. Like the imaginal discs that originate the hindlegs, the wing imaginal discs are also under the influence of differential levels of JH, which might be the cause of the caste-specific wing morphologies.

### The proportion of upregulated DEGs changes drastically in the wing discs of workers at the height of the metamorphic molt

We found that at the L5PP2 phase, the worker wing discs are distinguished by a higher proportion of upregulated genes than queens. Inversely, the proportion of genes upregulated in queen wing discs and descendant structures is higher in all the other developmental phases. The L5PP2 phase is a critical phase of metamorphosis, distinguished by a peak of ecdysteroids [20]. At this time, the wing discs take the shape of pupal wings and thoracic dorsum under the larval cuticle. The pharate-pupal development ends when the larval cuticle is discarded (ecdysis), thus revealing the pupa (PW phase) with its newly formed wings and thoracic dorsum. A new apolysis after the end of PW phase marks the beginning of adult cuticle synthesis, and subsequently, its pigmentation and sclerotization [21]. At adult ecdysis time, the wing disc-derived structures phenotypically differ between workers and queens (Additional file 14: Supplementary Figure 7) [35, 43]. Therefore, execution of the wing discs metamorphic program embodies differential gene expression between castes, which is more evident at the peak of metamorphosis (L5PP2 phase), and culminates with the wing-discs derived structures showing distinct phenotypes. Although not as evident as the presence/absence of wings in ant castes, differences in wing-disc derived structures do exist in *A. mellifera* castes. They are measurable and consistent with the observed differential gene expression.

### Variable expression profiles of functionally related DEGs

#### Ecdysis-related genes (except *Jhe*) were all upregulated in worker wing discs at the height of metamorphosis (L5PP2 phase)

Ecdysone-responsive genes, as those highlighted in our RNA-seq analysis, are part of a signaling cascade [47], which regulates metamorphosis and ecdysis in insects. In the honeybee, ecdysone (ecdysteroids) and JH show caste-specific levels [20, 22], which may explain the differential expression of ecdysis-related genes, as herein demonstrated. The gene encoding EcR showed a higher expression in the developing wing discs of workers than queens. In support, a higher expression was previously observed in whole-body samples of workers than queens [48]. As expected, the expression profiles of the genes encoding EcR and its partner, Usp, clustered together in the heatmap (Fig. 6a), with a clear higher expression of *Usp* in workers at the L5F1, L5PP2, and PW phases, which is also consistent with previous data, but using fat body of honeybee pupae and pharate-adults as samples [49].

Similarly, genes encoding molting-related transcription factors (*Br-c, E74*, and *ftz-f1*) and ecdysis-related peptides (*Eh* and *Eth*) showed higher expression levels in workers than queens, more specifically at the L5PP2 phase, when ecdysteroid titer peaks and wing discs undergo metamorphosis. However, *E74* and *ftz-f1* were reinduced in conditions of basal ecdysteroid levels at the PBD phase, suggesting ecdysteroid-independent roles in wings and thoracic dorsum at the proximity of adult ecdysis. A higher expression of *ftz-f1* in the fat body of the PBD phase, compared to earlier pharate-adult phases, was already described in the honeybee [50].

Consistent with the essential function of *Eh* in the control of ecdysis in *Drosophila* [51], the higher levels of *Eh* transcripts in the metamorphosing wing discs (L5PP2 phase) and early pharate-adult wings/thoracic dorsum (PB phase) of both honeybee castes preceded the ecdyses of the pupal and adult cuticle, respectively. Interestingly, the ecdysteroid titer that induces apolysis in L5PP2 queens is approximately twice that estimated for workers at the same developmental phase [20], but *Eh* expression level is lower in queens. Apparently, induction of *Eh* depends on a certain ecdysteroid level threshold, above which the hormone becomes inhibitory. The same reasoning may be behind the caste-differential expression of *Eth* and the above-mentioned ecdysis-related genes at the L5PP2 phase.

The gene *Jhe* is maximally expressed in the wing discs of workers at the L5F1 phase. This result is consistent with our previous work on JHE enzyme activity and its role in downregulating JH titer in worker feeding larvae [52]. Interestingly, we observed that *Jhe* was significantly less expressed in the wing discs of queens at this same developmental phase (L5F1), also consistent with the requirement of a high JH titer at this stage for queen caste determination [20] and, possibly, for caste-specific wings/thoracic dorsum differentiation. Like *Jhe*, the JH-epoxide hydrolase gene, *Jheh*, encodes a JH degrading enzyme. However, in the honeybee, this enzyme has a minor role, if any, in JH degradation [53]. Coherently, the expression patterns of *Jhe* and *Jheh* considerably differ in the imaginal discs and derived structures. The higher *Jheh* expression in workers than queens at the L5PP2 phase, when the JH titer is lower in workers, could even be intuitively related to an action of JH-epoxide hydrolase in lowering the hormone titer. However, these assumptions require experimental confirmation as *Jheh,* like *Jhe*, is also expressed at higher levels in queens than workers at the PBD phase, near the ecdysis to the adult stage, when JH titer is higher in queens [54].

In the L5F1 phase, JH titer is much higher in queens than workers, but subsequently, this difference is greatly minimized by JH decay to low levels in both castes in preparation for the metamorphic molt [20]. The decay in JH titer is an essential condition for the release of ecdysone by the prothoracic gland and wing imaginal discs growth. Components of the insulin signaling pathway are involved in this process [55, 56]. Insulin prevents the suppressive effect of JH on the expression of *broad* (a molecular marker of pupal commitment) and wing discs differentiation [12]. Consistently, *Br-c* is induced in the metamorphic phase (L5PP2), mainly in workers. Our libraries contain genes involved in regulating metamorphosis by insulin, such as insulin receptor substrate (*chico*), insulin-like peptide 2 [57], FOXO, and S6K.

#### Expression profiles of chitin- and melanization/sclerotization-related genes clustered separately (except for *laccase-21-like* gene) according to their functional characteristics

Other genes expressed in the wing discs and descendant structures were those related to chitinolysis and melanization/sclerotization, making feasible cuticle renewal and maturation. Chitin, the main structural polysaccharide in the cuticle, and melanin, which is necessary for pigmentation/sclerotization, are constituents of the cuticle covering the wings and thoracic dorsum. The products of chitinase genes catalyze the hydrolysis of chitin whereas the products of N-acetylglucosaminidases genes catalyze the specific hydrolysis of chitooligosaccharides [58]. We found in our RNA-seq libraries chitinase-encoding genes (*Cht*, *Cht2*, *Cht3*, *Cht5*) as well as the chitooligosaccharidolytic beta-N-acetylglucosaminidase encoding gene (LOC725178). All these genes showed distinct expression profiles during the studied developmental period, suggesting that they are opportunely required for renewal of specialized regions or structures of the cuticle. In fact, the downregulation of transcripts for chitinase genes of *T. castaneum* yielded quite different phenotypes [59]. Other chitin-related genes expressed in our libraries, the chitin-deacetylase genes (*Cda4*, *Cda5*), encode enzymes that deacetylate chitin to form chitosan that can potentially be bound by other proteins than those exclusively binding chitin. As for the chitinase genes, the differences in developmental profiles of *Cda4* and *Cda5* genes may indicate functional specialization. In addition, they showed caste-differential expression through wing discs development. Another gene included in the chitin-related group putatively encodes a chitinase-like Idgf4 protein (LOC724987) that lacked the catalytic site and thus the enzyme activity. The precise function of this protein in insects is unclear. Transcripts for some of the chitin-related genes here identified, *Cht3*, *Cht5*, *Cda4*, *Cda5*, and *Idgf4-like* (LOC724987), were also verified in the abdominal integument of honeybee pharate-adults [60].

As expected, genes related to the melanization/sclerotization pathway like *TyHyd*, *Dat*, *Ddc*, *Lac2*, and *Pxd* were induced later, when the cuticle lining the wings and thoracic dorsum is ready to start pigmentation (in PB phase) or is almost completely pigmented and hardened (in PBD phase). *Lac2*, for example, is known as involved in the cross-linking of cuticular proteins and quinones for cuticle sclerotization. It is upregulated in honeybee pharate-adults [39]. Surprisingly, another key gene involved in cuticle melanization/sclerotization in the honeybee, *PPO* [61], was expressed in the unpigmented wing discs of the L5F1 phase. Still, we also observed relatively high *PPO* expression in the pigmented wings/thoracic dorsum of workers at the PBD phase. The *laccase-21-like* gene was also upregulated in the pharate-adults (PB phase), consistent with a cuticular sclerotization function, but this was not observed for the other identified laccase genes *laccase-1-like* and *laccase-5-like*. Transcripts for the *Pxd* gene were previously identified in a cDNA microarray using honeybee integuments as samples (GB10387 in the 4.0 version of the honeybee genome [23]. The corresponding Pxd protein was validated (XP_006558162.1) [62] and further renamed as XP_016768434.2). As other genes involved in cuticle melanization and sclerotization, *Pxd* showed increased activity in the developmental phase before the appearance of pigments in the cuticle (PB phase, this work) and during cuticle pigmentation [23]. The other peroxidase genes here detected, *peroxidase* and *peroxidase-like*, also showed higher transcript levels prior (PB phase) and/or during (PBD phase) melanization/sclerotization, suggesting roles in these processes.

The chitin-related and melanization/sclerotization genes showed differential expression in workers and queens’ wing discs and descendant structures, thus highlighting caste-specific differences in their temporal expression curves. Interestingly, several melanization/sclerotization-related genes (*ebony*, *peroxidase*, *TyHyd*, *Dat*, *Ddc*, *Lac2*, and *Pxd*) showed a higher expression in queens than workers in the developmental phases where they were more expressed. *TyHyd*, which encodes the enzyme catalyzing the first step in the melanin synthetic pathway, was strongly induced in the PBD phase that precedes adult ecdysis. At this stage wings and thoracic dorsum are already melanized and quite sclerotized [21], and the higher expression of *TyHyd* may be related to the intensification of pigmentation and hardening of the cuticle.

#### Cuticular protein genes of the CPR, Tweedle, CPF, CPLCP, CPAP, and Apidermin families are expressed in wing discs and derived structures

The CPR class genes *AmelCPR13* and *AmelCPR21* were maximally expressed in the premetamorphic wing discs of the L5F1 worker and queen larvae. We also observed upregulation of *AmelCPR1*, *AmelCPR2*, and *CPF1*, with maximal expression in the wing discs of workers, during apolysis of the larval cuticle and onset of pupal cuticle synthesis at the L5PP2 phase. Certainly, these genes are involved in the pupal cuticle synthesis. In contrast, *Apd-1* and *Apd-3* genes were upregulated in the wing discs exclusively at the proximity of adult ecdysis (PBD phase), consistent with our previous data on *A. mellifera* integument transcriptome [60], suggesting functions in the structure of the pre-ecdysial adult cuticle. Transcripts for the five genes of the CPAP family in the honeybee are represented in the transcriptomes of wing discs and derivatives. Three of these genes (*Cpap3-b*, *Cpap3-c*, *Cpap3-d*) clustered closely on the heatmap, thus revealing similar expression profiles, and perhaps, similar roles that possibly differ from the other two genes in this family (*Cpap3-a*, *Cpap3-e*).

We also observed conspicuous caste-differential expression of some of these cuticular protein genes. As observed for other genes in the heatmaps, they change from a higher expression in workers at a particular developmental phase to a higher expression in queens at another phase and vice-versa. For example, *AmelCPR14* was more expressed in workers than queens at the L5PP2 phase, and more expressed in queens than workers at the PB and PBD phases, indicating caste-specificity in expression profile dynamics. However, genes pertaining to a same class were induced in both castes at the same developmental phase, although their expression profiles don’t match exactly. For example, *AmelTwdl-2* was maximally expressed in L5PP2 in workers and queens, and so we observed for the other Tweddle-family gene, *AmelTwdl-1*, suggesting similar roles in wing discs at the height of metamorphosis. Likewise, the genes *CPLCP1* and *CPLCP2* showed both the highest expression in the PB phase, in queens and workers. The structural features that allowed classification of the cuticular proteins, and their genes, into distinct families have been reviewed in [63].

#### Wing disc metamorphosis in the honeybee involves the expression of fourteen *Drosophila* wing patterning orthologs

In the honeybee transcriptome profile datasets, we searched for putative orthologs of *Drosophila* genes participating in the wing patterning network, as illustrated in [36]. One of these genes, *vg*, is crucial for wing and haltere formation in *Drosophila* [64]. Its strongest expression in wings/thoracic dorsum of pharate adults evidences its role in the final steps of formation of these structures. In *Drosophila*, the products of *vg* and *sd* function coordinately to regulate the expression of wing development genes [65]. However, in *A. mellifera, sd* and *vg* expression profiles differed from each other, and a partnership between their respective proteins could not be intuitively deduced.

In *Drosophila*, the induction of *vg* in wing discs requires the activities of *Ser* and *wg* [66]. *Ser* activity is needed for wing blade correct development [67], and *wg* function is crucial to distinguish wing/notum fields in the wing imaginal discs [68]. Wing discs of *B. mori* express the *wg* ortholog, *Wnt1* [69], which we also detected in our honeybee libraries. We observed that the expression patterns of *Ser* and *Wnt-1* clustered together in the heatmap, suggesting a coordinate action, but *vg* expression pattern clustered separately. *Omb* is another gene required for activation of *vg* in *Drosophila*. It is expressed in the wing discs of larvae, and also in pupae [70], like in the honeybee. As we could see, *omb* expression decays in pharate adults (PB and PBD), in contrast to *vg* expression, suggesting that *omb-vg* interaction does not occur in wings/thoracic dorsum at these developmental phases.

Interestingly, the expression profile dynamics of *vg* and *how* clustered together, with maximal activity in queens and workers at the PB and PBD pharate-adult phases, suggesting that like *vg*, *how* has roles in wings/thoracic dorsum maturation. In *Drosophila*, the expression of *how*, also known as *who*, was described in muscle cell precursors attached to the wing imaginal disc [71]; *how* also may have roles in imaginal disc eversion [72]. As we are here demonstrating, *how* is also strongly expressed after metamorphosis in the body structures derived from the wing discs.

The expression of the wing patterning genes *Ubx* and *ct* have already been demonstrated throughout immunohistochemistry in the forewing discs as well as in the hindwing discs of fifth instar honeybee larvae [73]. Consistently, we found *Ubx* and *ct* expression in our wing disc samples, each prepared with fore- and hindwing discs pooled together. We verified that the expression patterns of *Ubx* and *cut* clustered together in the heatmap, thus evidencing similar gene expression modulation.

The gene *mwh* showed a relatively low expression in the honeybee wing discs and their descendants. In *Drosophila*, *mwh* has been related to polarized hair formation on the distal side of the wing cells [74]. Compared to the other wing-forming genes, *mwh* expression was more uniform through honeybee wing discs development, which does not allow us to establish a relationship, even intuitive, between *mwh* transcript levels and the surge of cuticular hairs in wings and thoracic dorsum.

The other two genes that interact in *Drosophila* wing discs are *hh* and *En*. The gene *hh* provides positional information under the control of *En*, which specifies the posterior compartment of the wing [75]. In our samples, *hh* and *En* transcripts displayed moderate to low but distinct expression level profiles. Their respective expression patterns are more similar to each other in the premetamorphic wing discs of the L5F1 phase, which may suggest interaction for wing disc patterning.

In *Drosophila, exd* is necessary for the patterning of notum structural elements and is expressed in wing discs and also during notum development in pharate adults [76]; *exd* expression was detected in the developing forewing and hindwing buds of the honeybee [73] and was here confirmed in their derivatives, with the highest expression after the metamorphic molt (PW phase). The gene *ap* encodes a transcription factor involved in major wing dorsal-ventral patterning events in *Drosophila* [77]. This gene is expressed at different levels in pea aphid’s winged and unwinged morphs, suggesting roles in polyphenic development [78]. Consistently*, ap* modulates wing patterning, size, and bristle formation in the long-winged and short-winged morphs of the hemipteran *Nilaparvata lugens* [79]. Further studies may clarify whether *ap* has a similar role in differentiating wings in the honeybee castes.

In *Drosophila*, the *wingless* paralog *Wnt6* is expressed at the dorso-ventral boundary, hinge, and notum-destined region of the wing discs, in a pattern comparable to that of *wingless* [80]. *Wnt6* is needed for the correct positioning and spacing of chemosensory bristles in the wing margin [81]. In the honeybee castes, the *Wnt6* gene is differentially expressed in the premetamorphic and metamorphic wing discs and pupal wings/thoracic dorsum. Whether caste-differential expression of *Wnt6* and the other wing-patterning orthologs are related to caste-specific phenotypes is a matter for further studies.

In addition to the classes of genes discussed above, we emphasize here that genes for odorant binding proteins (OBPs) were also expressed in the wing discs and their descendants. Odorants and pheromones are recognized by OBPs that provide their delivery to olfatory receptors (ORs) in the olfactory sensilla, thus triggering the signal transduction toward the insect brain and, consequently, the appropriate response [82]. OBPs, however, are not exclusive of olfactory tissues, like antennae, where the majority of olfactory neurons reside but have also been found in accessory sex glands of *T. molitor* [83], mandibular glands of *A. mellifera* [84], and the mandibular region of *Melipona scutellaris* larvae [85].

The genome of the honeybee contains 21 *Obp* genes [86], and 10 of them were expressed in the wing discs and/or derived structures herein analyzed, where they may perform roles other than the canonical one. In support of this assumption, we detected only two OR gene forms (*13a-like1* and *13a-like2*), expressed in basal levels, from a total of 177 OR genes in the honeybee genome [87]. In the wing discs and derivatives, the *Obp* genes showed differential expression between the developmental phases (*Obp14* and *Obp18* are maximally expressed in the L5F1 and PBD phases, respectively) and between castes (*Obp15* is significantly more expressed in L5F1 queens) (Additional file 15: Supplementary Figure 8). Expression of *Obp* genes was also found in the wings of two other hymenopterans, *Polystes dominulus* and *Vespa crabro* [88], and in the legs and thorax of *A. mellifera* [86]; all these body structures derive from imaginal discs.

### Ame-miRNAs putatively involved in wing disc metamorphosis

In the small RNA libraries of wing buds (L5PP2 phase), we found ame-miRNAs whose homologs in other insect species are known as having roles in metamorphosis. Ame-miR-100-5p and ame-miR-125-5p are the second and third more expressed ame-miRNAs, and ame-let-7-5p is the thirteenth most expressed. The 3p arm of each of these miRNAs also showed abundant, although lower, processing in our libraries (Additional file 11: Supplementary Table 7). Like their homologs in most insect species [89], these miRNAs cluster together in the same primary transcript. They are located *in tandem* in the linkage group LG8 of the honeybee genome. In *Drosophila*, let-7 and miR-125 regulate wing morphogenesis: both are critical for the appropriate timing of cell cycle exit, cessation of cell division, and wing imaginal discs differentiation [90, 91]. In *B. germanica*, let-7, miR-125, and miR-100 showed increased expression in wing pads, and depletion of let-7 and miR-100 provoked wing size and vein patterning defects [92, 93]. We can hypothesize correlated functions for the homolog ame-miRNAs.

Other miRNAs considered important for wing morphogenesis in *B. mori* [94] are members of the miR-2 family (miR-2, miR-13a, miR-13b). In our libraries, the miR-2 family is represented by ame-miR-2-1-3p, ame-miR-2b-5p, ame-miR-2-3-3p, ame-miR-2-2-3p, which are among the top 20 more expressed miRNAs, and by the less expressed members ame-miR-2-2-5p and ame-miR-2-3-5p; the ame-miR-13b-3p and ame-miR-13a-3p members are among the 30 more expressed miRNAs, with the opposite arms (ame-miR-13b-5p and ame-miR-13a-5p) being less expressed. In *Drosophila*, the miR-9 family has also been related to wing morphogenesis [95]. The honeybee wing discs express ame-miR-9a-3p, ame-miR-9a-5p and ame-miR-9b-3p. The number of reads places them among the 50 more expressed ame-miRNAs; ame-miR-9b-5p is less expressed. The relatively high expression levels of these ame-miRNAs suggest that, like in *Drosophila*, they display functional activity in the metamorphosing wing discs of the honeybee.

There are ame-miRNAs whose homologs have not explicitly been related to wing morphogenesis but the larval-to-pupal transition. In *B. mori*, for example, miR-31a, miR-275, and miR-276 are more expressed prior to the metamorphic molt in spinning larvae.Then their levels decrease in abundance in pupae [96], suggesting roles in larval-to-pupal transition. These miRNAs were also abundant in *B. gemanica* last nymphal stage, which is the metamorphic stage, similarly suggesting roles in metamorphosis [97]. In the honeybee, ame-miR31a-5p, ame-miR-275-3p and ame-miR-276-3p, may have similar roles in metamorphosis, standing out that ame-miR-275-3p and ame-miR-276-3p occupy the fourteenth and fifth position, respectively, in the ranking of more expressed ame-miRNAs; ame-miR-31a-5p ranks 21 in our list. Worthy of mention, most of the miRNAs expressed in the metamorphic stage of *B. germanica* [98] are represented by homologs in our libraries of metamorphosing honeybee wing discs.

All the ame-miRNAs identified here are expressed in synchrony with the ecdysteroid pulse [20] in the L5PP2 developmental phase. Homologs of some ame-miRNAs in other insect species [91] are known as involved in 20E-signaling pathway. In *Drosophila*, expression of let-7, miR-125, miR-100, and miR-34 during metamorphosis requires ecdysone [99, 100], and miR-8 [101] and miR-252 [102] are both regulated by 20E. In *B. mori*, the expression of let-7 has also been correlated to ecdysteroid pulses [103]. The ame-miR-965 homolog in *Drosophila* is inhibited by ecdysone, leading to increased histoblasts proliferation during metamorphosis. In turn, this miRNA reduces the level of ecdysone receptor [104]. Also, there is evidence that bantam, which is highly expressed in our honeybee libraries, and other miRNAs, are involved in regulating ecdysteroid biosynthesis in the holometabolous insect *Chilo suppressalis* [105]. Furthermore, homologs of ame-miR-14 in *Drosophila* [106], *B. mori* [107], and *C. suppressalis* [108] were related to metamorphosis regulation by interfering in the 20E signaling pathway. We can suggest a correlating role for ame-miRNA-14-3p and ame-miRNA-14-5p in the developing wing discs of the L5PP2 phase. Similarly, ame-miR-281 possibly has functions in the 20E signaling pathway, like observed for its homolog in *B. mori* [109].

Based on information obtained from homologs in other insect species, functional activities other than involvement in metamorphosis can also be hypothesized for the highly expressed ame-bantam-3p and ame-miR-8-3p miRNAs, and for an ame-miRNA represented by a relatively low number of reads, ame-miR-252a-5p. In *Drosophila*, bantam controls body size by linking the insulin pathway and ecdysone production [110]; miR-8 controls body size [101] and has been related to the regulation of growth factors in the fat body [111]. In *B. germanica,* miR-252 is involved in general growth and development [98]. Furthermore, both ame-miR-277-3p and ame-miR-278-5p may have orthology relationships with the *Drosophila* miRNAs displaying roles in lifespan (miR-277) [112] and energy homeostasis (miR-278) [113].

We did not find metamorphosis-related functional activities, experimentally confirmed or even predicted, for homologs of ame-miR-10, whose 5p arm is the top one in our ranking of more expressed ame-miRs (almost 20 million reads). The same observation applies to some ame-miRNAs among the top 20 highly expressed in our list. Further investigations are required to elucidate their roles in the honeybee wing discs.

We also focused on the targets of those ame-miRNAs whose homologs in other insects have been related to wing development or metamorphosis. Among the DEGs downregulated in the developing wings of the L5PP2 phase, a few displayed complementarities with ame-miR-100-5p or ame-miR-125-5p. Ame-let-7-5p targets several DEGs; however, the majority of them have no orthology relationships with genes from other insect species (see Additional file 12: Supplementary Figure 5). Interestingly, ame-let-7-5p targets *def-1* transcripts that encode an antimicrobial peptide important for the immune response in the honeybee [114]. *def-1* transcripts are also putative targets of ame-miR-14-5p and ame-miR-34-5p. The involvement of miRNAs in the immune system has been described in insects. In *Drosophila*, for example, the antimicrobial peptide gene *diptericin* contains a binding site for translation repression by let-7 [115]. In the honeybee, ame-let-7 interacts with genes in the broadly-conserved NF-κB immune signaling pathways, IMD (Immune Deficiency), JNK (Jun-N-terminal Kinase), and Toll [116], thus regulating the production of antimicrobial peptides for cellular defense response. Similarly, ame-bantam interacts with transcripts encoding the antimicrobial peptide hymenoptaecin [117] and targets the mRNA encoding Vg, the multifunctional egg protein involved in the immune response [118]. Like ame-bantam, ame-miR-9b-3p and ame-miR-252b-5p both have *hymenoptaecin* mRNA as a target, and ame-miR-34-5p has *Vg* mRNA as a target, suggesting negative regulation in workers by miRNAs*.*

Interesting putative targets of miRNAs expressed in the L5PP2 wing discs are *TyHyd* (targeted by ame-miR-14-5p and ame-miR-252a-5p)*, Lac2* (targeted by ame-miR-276-3p, ame-miR-2b-5p, ame-miR-281-3p, ame-miR-14-5p and ame-miR-34-5p) and *PPO* (targeted by ame-miR-281-3p and ame-miR-34-5p). Although these interactions require experimental confirmation, the negative regulatory roles of these ame-miRNAs are supported by the observed lower levels of *TyHyd* and *Lac2* transcripts before, during, and immediately after the larval-pupal transition (L5F1, L5PP2, and PW phases). Likewise, *PPO* transcript level is lowered at the height of metamorphosis (L5PP2 phase) and remains low to only increase near the adult emergence (PBD phase), in workers (Fig. 6b). In an ecdysteroid titer-dependent manner, *TyHyd* [119]*, Lac2* [39], and *PPO* [120] genes are expressed in the epidermis of developing honeybees where they have critical roles in adult cuticle differentiation and certainly in the differentiation of the wing cuticle.

## Conclusion

In conclusion, we described the dynamic transcriptional scenery encompassing the period of wing discs metamorphosis to wings and thoracic dorsum in the honeybee. Transcriptomes profiling through RNA-seq technology revealed the global gene expression needed for building adult body parts from primordial larval structures. The results also underscored miRNAs expressed in the wing buds at the height of metamorphosis and predictive interactions with mRNAs. The obtained datasets disclosed the molecular basis of wing discs metamorphosis in a two-winged model insect, paved the way for further functional studies (through fluorescence in situ hybridization, immunohistochemistry, and gene silencing mediated by RNAi, among other methods), and certainly will contribute to comparative and evolutionary approaches. Our analyses also revealed differential gene expression in the metamorphosing wing discs of the honeybee castes.

## Methods

### Sample collection

Africanized *A. mellifera* workers and queens were collected from three colonies originated from different places in the São Paulo state, Brazil: Experimental Apiary of the Medical School in Ribeirão Preto, São Paulo University (Latitude: 21° 10′ 39“ S; Longitude: 47° 48’ 37” W), and apiaries in the cities of Luiz Antônio (Latitude: 21° 32′ 58″ S; Longitude: 47° 42′ 24″ W) and Atibaia (Latitude: 23° 07′ 01“ S; Longitude: 46° 33’ 01” W). All colonies were maintained in the Experimental Apiary in Ribeirão Preto during sample collection.

Developing workers and queens were staged [19, 24] and collected at the onset of the last (fifth) larval instar (L5F1 feeding phase), at the end of this instar (L5PP2 pharate-pupae phase), immediately after pupal ecdysis (unpigmented cuticle, white-eyed PW phase), at an early pharate-adult phase (unpigmented cuticle, brown-eyed PB phase), and at the late pharate-adult phase (pigmented cuticle, brown-eyed PBD phase) (See Additional file 1: Supplementary Figure 1 and Additional file 2: Supplementary Table 1 for characteristics of staged bees). The developing workers were collected directly from brood frames. Queens were produced through standard beekeeping techniques [121] and collected at the developmental phases specified above.

### Dissection of wing discs and derivatives for mRNA sequencing

After collection, the developing workers and queens were dissected for extraction of the wing discs (L5F1 samples), wing discs in metamorphosis (L5PP2 samples), and wing disc descendants structures, i.e., wings plus thoracic dorsum integument of pupae (PW samples) and pharate-adults (PB and PBD samples). Dissection was rapidly done in sterile 0.9% NaCl solution. We prepared three independent biological samples (triplicate) for each of the five developmental phases of workers and queens, in a total of 30 samples. Each of the three L5F1 samples was prepared with wing discs (fore- plus hindwing disc pairs) dissected from 30 worker larvae or 30 queen larvae. Each L5PP2 sample was prepared with metamorphosing wing pairs (fore- plus hindwings) dissected from 5 workers or 5 queens. Each PW, PB, and PBD sample was prepared with wings (fore- plus hindwings) attached to the thoracic dorsum from 5 workers or 5 queens. To ensure a greater variability, each sample in the triplicate was prepared with wing discs or descendant structures taken from workers or queens from one of the three different colonies. Samples were stored for a short time at -80 °C before total RNA extraction.

RNA extractions were made using TRIzol reagent (Invitrogen) following the manufacturer’s instructions. The purity and concentration of RNA extracted from each sample were determined through optical absorbance at 260/280 nm using NanoDrop ND-1000 (NanoDrop Technologies). The RNA samples (2 μg/per sample) were sent to a facility (Laboratório Central de Tecnologias de Alto Desempenho em Ciências da Vida, LaCTAD, Universidade Estadual de Campinas, Campinas, Brazil) to assess sample quality using a 2100 Bioanalyzer, and for libraries preparation using TruSeq RNA sample preparation (Illumina) and a system of adaptors. RNA samples were sequenced in an Illumina HiSeq 2500 equipment (single-end reads, 2 × 100 bp read length). We obtained an average of 30 million reads per sample, with 90% of the nucleobases showing quality scores *>* Q30. The RNA-seq data are deposited at the National Center for Biotechnology Information (NCBI) database under the BioProject ID PRJNA724861.

### Adapters trimming and quality check

The software bcl2fastq v.1.8.4 and default parameters were used for the removal of adapter sequences. The poor-quality reads and poli (A/T) tails were removed by trimming using the software PRINSEQ-lite v. 0.19.5 [122], and sequence quality was evaluated through the software FastQC v. 0.11.2 [123]. We filtered out low-quality sequences (reads with Phred quality < 25). Reads less than 15 nucleobases in length or containing more than 80% of ambiguous nucleobases (N) were discarded.

### Transcriptome assembly, gene expression, and predictive gene functions

We aligned the high-quality reads against the *A. mellifera* genome v. 4.5 [124, 125] using the software TopHat v. 2.0.7 [126]. We used the Amel 4.5 genome version in order to match the annotation of microRNAs retrieved from miRBase (release 22.1), which is based on this honeybee genome version. Reference gene coordinates used in the alignments were obtained in the NCBI (RefSeq v. 55) [127]. TopHat was set up prioritizing sensibility and precision (−-b2-very-sensitive). Alignment was performed with 20 nucleotides segments (−-segment-mismatches 2 --segment-length 20) allowing a maximum of two mismatches in the alignment (−-read-mismatches 2), with a maximum of three nucleotides for insertion or deletion (−-max-deletion-length 3 --max-insertion-length 3) and intron length between 20 and 200,000 bases in the exon junctions (−-min-intron-length 20 --max-intron-length 200,000).

After sequence alignment, the abundance of transcripts was determined using the software Cufflinks v. 2.1.1 [128, 129]. Transcript abundance was given in FPKM (Fragments Per Kilobase of transcript per Million mapped reads) using Upper-quartile [130] as the normalization method. The following parameters were enabled for tuning the Cufflinks pipeline: --multi-read-correct, −-frag-bias-correct, and--total-hits-norm. The extensions Cuffmerge integrated the reads to the mapping results, and Cuffdiff gave the expression levels for each sample and the significance of comparisons between samples. CuffmeRbund R package v. 2.8.2 allowed us to access all this information [131]. The results were filtered using an FDR adjusted *p*-value (or q-value) ≤ 0.05 and logFC ≥1 and ≤ − 1. Low FPKM values (< 5) were filtered out from all RNA-seq data.

All heat maps were designed using the function heatmap.2 from gplots R package [132]. For all groups of genes, we measured the clustering potential of the samples for each developmental phase and castes. For this approach, we used the R package pvclust v. 1.3.2 based on correlation distances, with a complete linkage method and 10,000 bootstrap replication. We used unbiased *p*-values (AU) and bootstrap values as measurements of clusters’ significance. Clusters showing AU *>* 95% were considered statistically significant [133].

DAVID (Database for Annotation, Visualization, and Integrated Discovery) was used for Gene Ontology (GO) functional analysis. The p-values from gene enrichment in GO annotation terms were adjusted using the Benjamini-Hochberg method with a false-discovery rate (FDR) < 0.05. Reactome pathway database (https://reactome.org) was used to find the most enriched biological pathways between and within the two honeybee castes. A combination of p-value and FDR correction < 0.05 was used to screen the enriched pathways.

Orthology relationships of 14 *A. mellifera* genes and *D. melanogaster* genes known as having roles in wing discs patterning were verified through Reciprocal Best Hit, a gold-standard method used to identify ortholog sequences. In this case, *A. mellifera* protein sequences encoding these genes were retrieved from the Beebase repository [125], and *D. melanogaster* protein sequences were retrieved from Flybase (version r6.40). These honeybee protein sequences were aligned against fruit-fly protein sequences and vice-versa using aligner diamond implemented in the Perl script getRBH.pl [134]. Only reciprocal ortholog pairs with coverage > 60% were considered for this analysis.

### Small RNA library preparation and computational analysis

We used wing discs in metamorphosis dissected from workers at the L5PP2 phase to prepare two samples, one containing 130 pairs of forewing discs and the other made with the same quantity of hindwing discs. Total RNA was extracted following Trizol manufacture’s protocol, and samples were used as templates in the preparation of small RNAs libraries, according to Illumina single-end protocol (Genome Analyzer II, Life Sciences). The libraries preparation and sequencing were performed at the High-Throughput Sequencing Facility in the University of North Carolina (Chapel Hill, USA). The libraries generated are deposited in www.ncbi.nlm.nih.gov/sra under access numbers SRR14319161 (forewing) and SRR14319162 (hindwing).

Computational analysis of small RNA libraries was conducted as described [135]. In summary, low-quality reads and adapter sequences were removed using Cutadapt software [136]. Filtered and trimmed reads were mapped to the honeybee genome (version amel 4.5) [125] using BWA [137]. The identification of expressed miRNAs was done by comparing reads and primary microRNAs coordinates (GFF file) using *bedtools* [138]. This approach led to identifying peaks of reads in both 5p and 3p arms of primary miRNA sequences, corresponding to both mature miRNAs, miRNA-5p and miRNA-3p. To adjust these miRNA data to the mRNA-seq data obtained from wing disc samples of workers (L5PP2 phase), made with fore- and hindwing discs pooled together, we considered the expression of each miRNA as the sum of read numbers of both miRNA libraries.

### Prediction of miRNA regulatory sites in the 3′ untranslated regions

We searched for miRNA regulatory elements (MREs) in the 3′ untranslated region of the DEGs identified in queens and workers at the L5PP2 phase using RNAhybrid [139] (free energy < − 20 kcal/mol and *p*-value < 0.05) and considering a region of 1000 bp downstream of the stop codons. Sequences of miRNAs were retrieved from miRBase (version 22.1) [140] and from the sequencing (this work) and used for target prediction analysis. This approach has successfully found functional MREs, as demonstrated [135]. To search for miRNAs whose targets were significantly enriched between the DEGs, we applied an enrichment score [141]. Putative miRNA-target networks were built using Cytoscape platform [142].

## Supplementary Information

**Additional file 1.**

**Additional file 2.**

**Additional file 3.**

**Additional file 4.**

**Additional file 5.**

**Additional file 6.**

**Additional file 7.**

**Additional file 8.**

**Additional file 9.**

**Additional file 10.**

**Additional file 11.**

**Additional file 12.**

**Additional file 13.**

**Additional file 14.**

**Additional file 15.**

## Data Availability

The RNA-seq and small RNA libraries datasets generated and analyzed during the current study are available at the National Center for Biotechnology Information (www.ncbi.nlm.nih.gov/sra). Access numbers: BioProject ID PRJNA724861 (RNA-seq libraries); SRR14319161 (forewing small RNA library) and SRR14319162 (hindwing small RNA library).
