# Supplementary figures and images for: Transcriptome dynamics during metamorphosis of imaginal discs into wings and thoracic dorsum in Apis mellifera castes
Source: BMC Genomics. 2021 Oct 22;22:756. doi: 10.1186/s12864-021-08040-z (PMC8532292; doi:10.1186/s12864-021-08040-z)

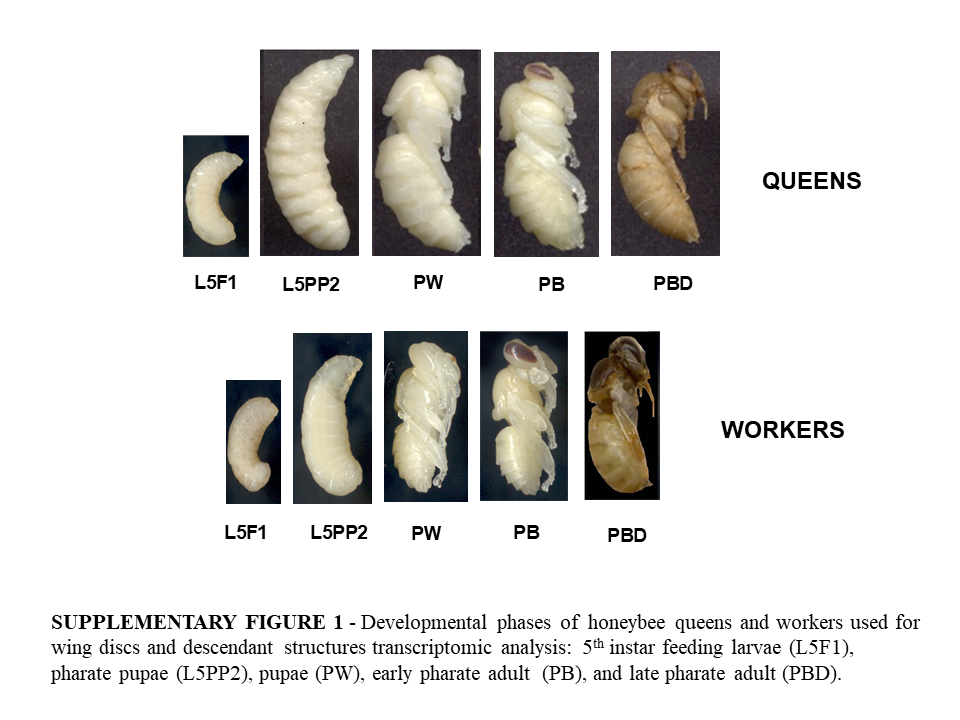

Supplement: Supplementary file 1 — Additional file 1. [file 12864_2021_8040_MOESM1_ESM.png]

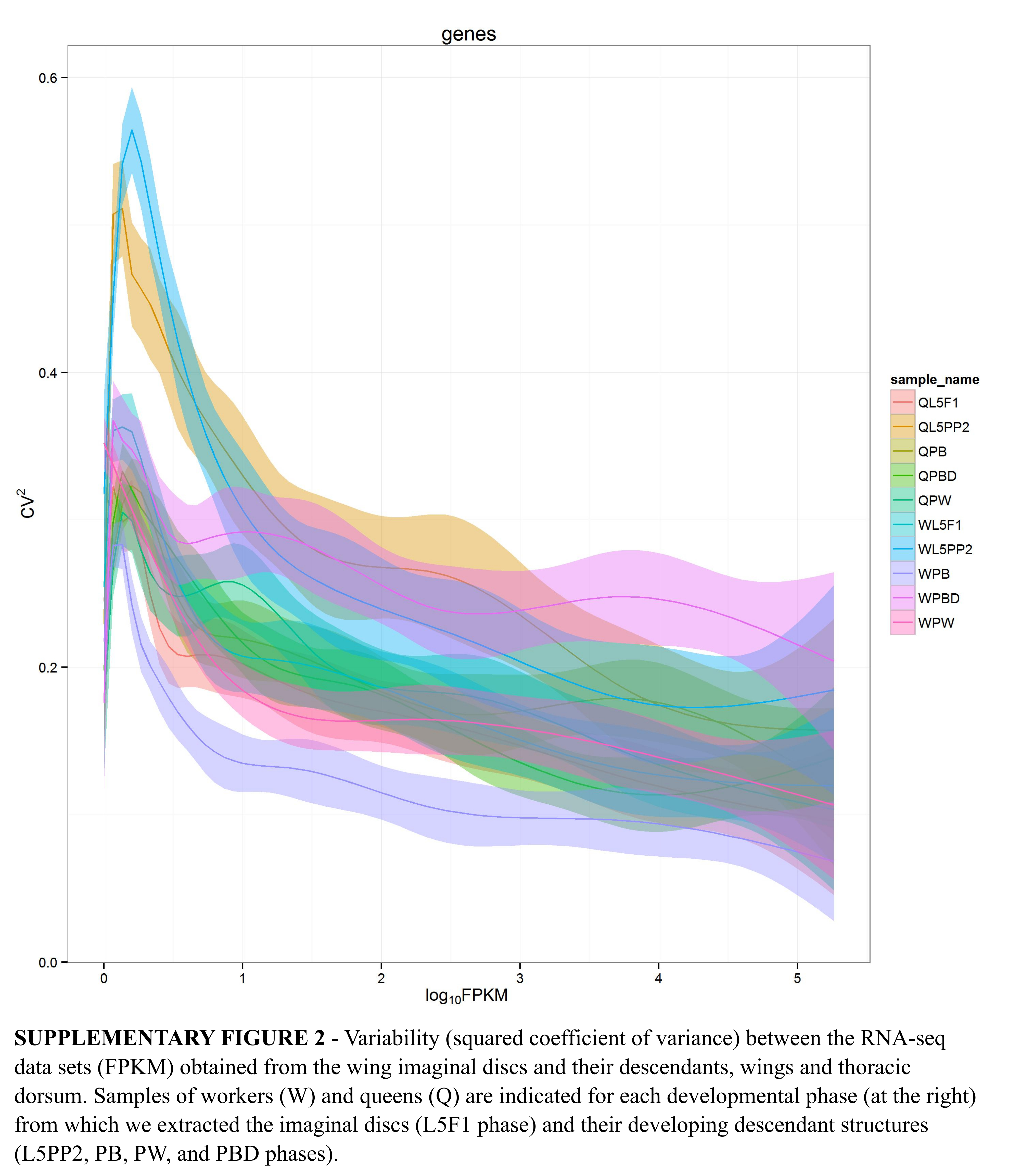

Supplement: Supplementary file 3 — Additional file 3. [file 12864_2021_8040_MOESM3_ESM.jpg]

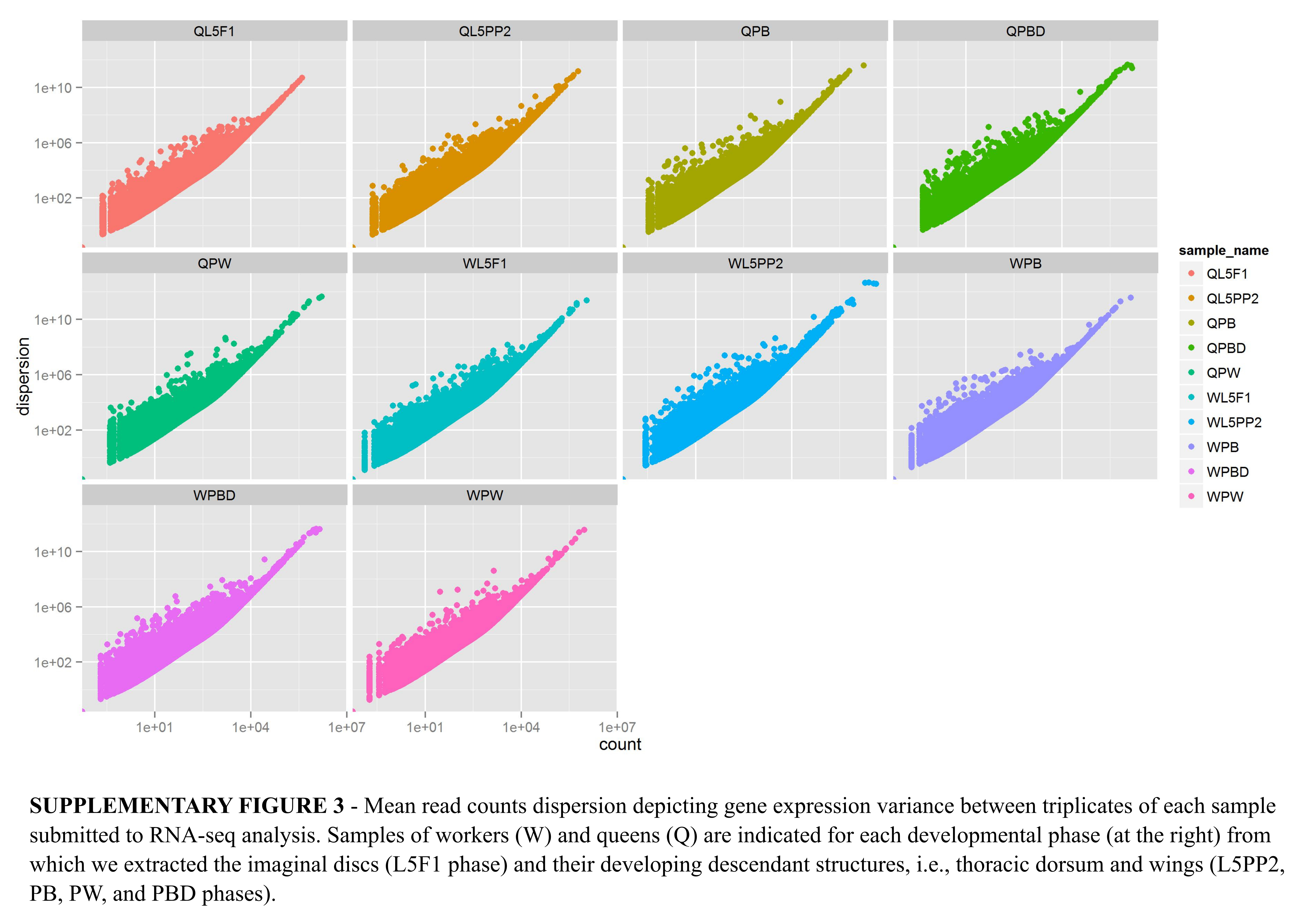

Supplement: Supplementary file 4 — Additional file 4. [file 12864_2021_8040_MOESM4_ESM.jpg]

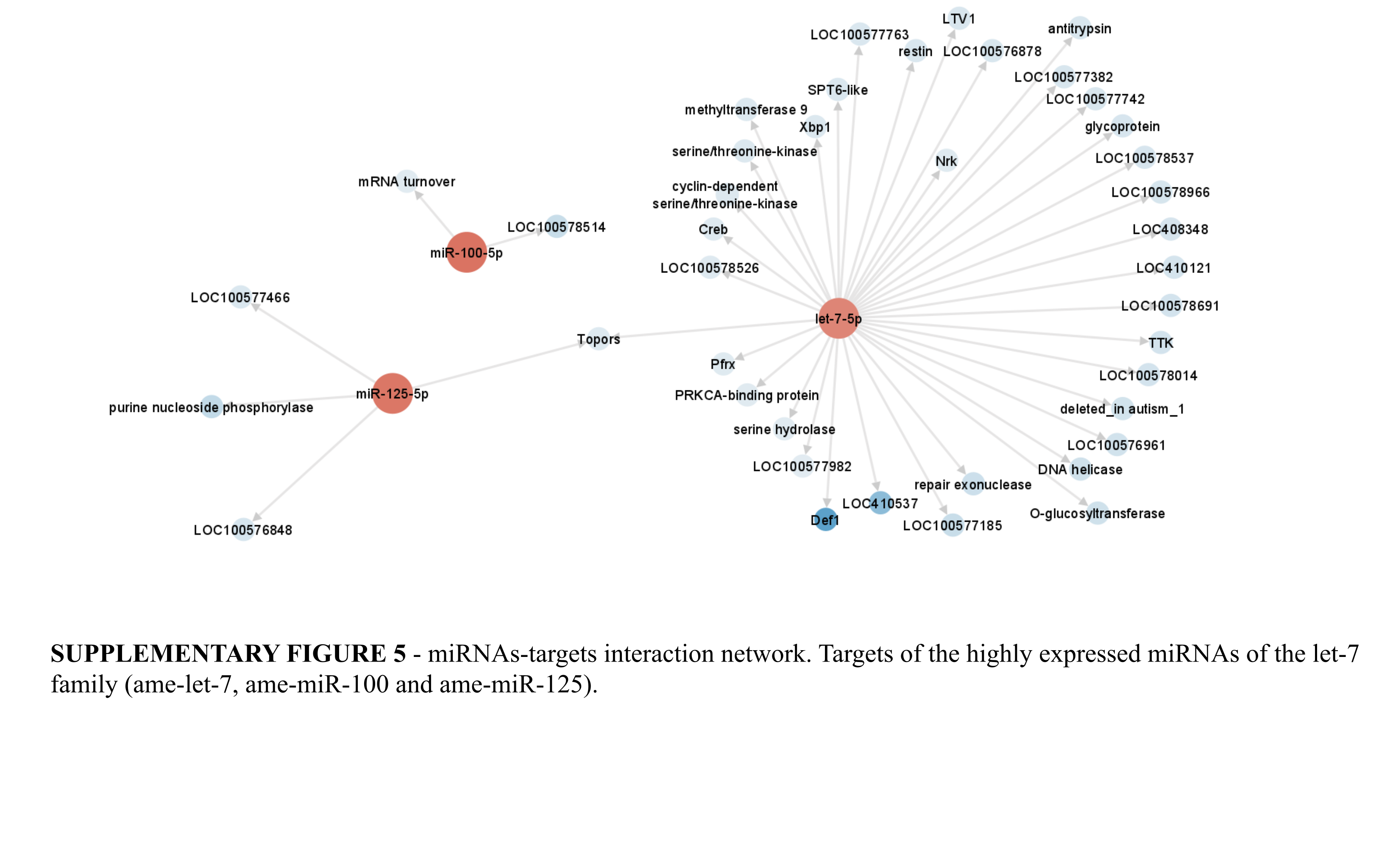

Supplement: Supplementary file 12 — Additional file 12. [file 12864_2021_8040_MOESM12_ESM.png]

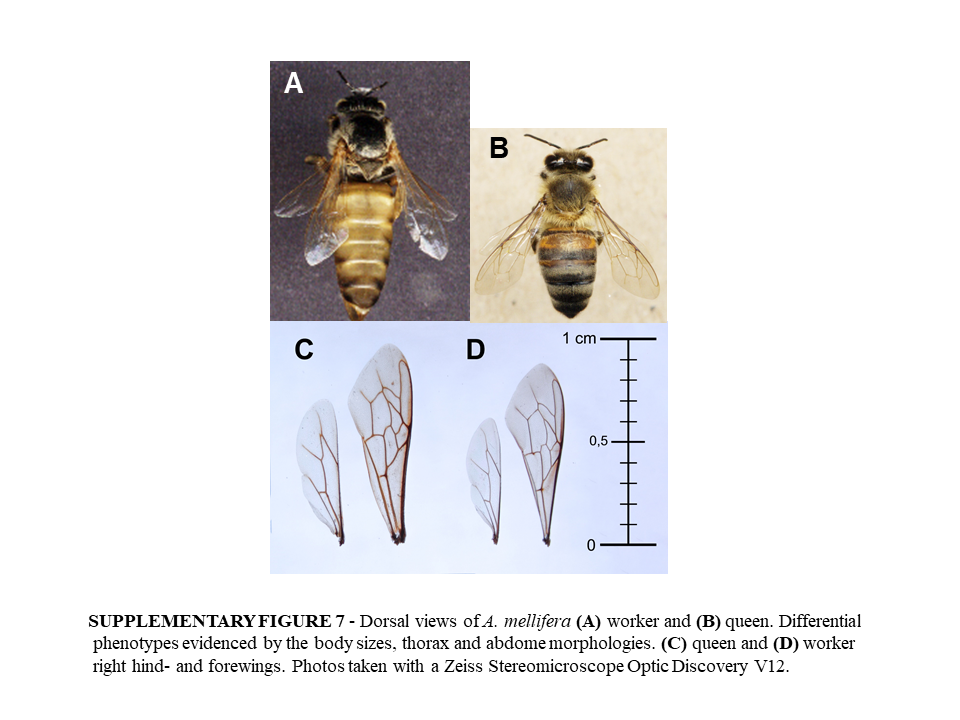

Supplement: Supplementary file 14 — Additional file 14. [file 12864_2021_8040_MOESM14_ESM.png]

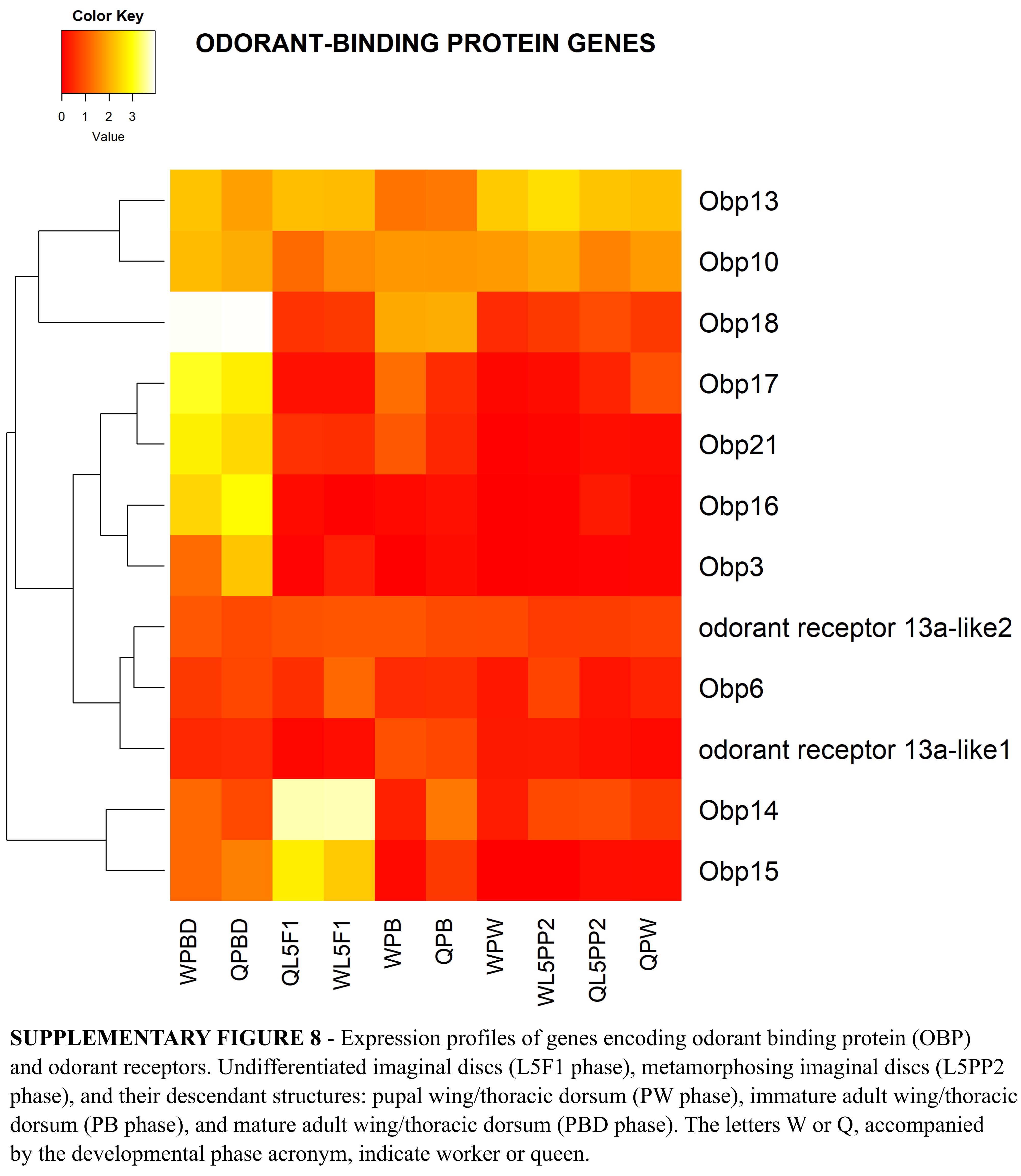

Supplement: Supplementary file 15 — Additional file 15. [file 12864_2021_8040_MOESM15_ESM.png]
