# Supplementary material for: Transcriptome dynamics during metamorphosis of imaginal discs into wings and thoracic dorsum in Apis mellifera castes
Source: BMC Genomics. 2021 Oct 22;22:756. doi: 10.1186/s12864-021-08040-z (PMC8532292; doi:10.1186/s12864-021-08040-z)
Supplement: Supplementary file 2 — Additional file 2. [file 12864_2021_8040_MOESM2_ESM.docx]

**Supplementary Table 1** – Description of external morphology and chronological age of developing queens (Q) and workers (W) collected for dissection of imaginal wing discs and their descendant structures (wings and thoracic dorsum). (-) weight measurements not used for developmental phase identification.

| **Developmental phases** | | **Castes** | **Age**  **(days)** | **Weight**  **(g)** | **Characteristics** |
| --- | --- | --- | --- | --- | --- |
| L5F1 | Fifth instar feeding larva | Q | 7 | 0,026 - 0,063 | The earlier phase of the fifth larval instar when larva is still feeding |
|  |  | W | 7 | 0,027 - 0,042 |  |
| L5PP2 | Pharate-pupa (prepupa) | Q | 10 | - | Pharate-pupa undergoing apolysis at the onset of the metamorphic molt |
|  |  | W | 11 |  |  |
| PW | White-eyed pupa, unpigmented cuticle | Q | 11 | - | Newly-ecdysed pupa |
|  |  | W | 13 |  |  |
| PB | Bro Brown-eyed pharate adult, unpigmented cuticle | Q | 14 | - | Early pharate-adult |
|  |  | W | 16 |  |  |
| PBD | Brown-eyed pharate-adult, dark  pigmented cuticle | Q | 16 | - | Late pharate-adult (the last pharate-adult phase that antecedes adult ecdyse) |
|  |  | W | 19 |  |  |

Developmental phase identification based in **Rembold (1987) [18], Michelette and Soares (1993) [19], and Nunes-Silva et al. (2006)** **[24]**.
