# Supplementary material for: Transcriptome dynamics during metamorphosis of imaginal discs into wings and thoracic dorsum in Apis mellifera castes
Source: BMC Genomics. 2021 Oct 22;22:756. doi: 10.1186/s12864-021-08040-z (PMC8532292; doi:10.1186/s12864-021-08040-z)
Supplement: Supplementary file 5 — Additional file 5. [file 12864_2021_8040_MOESM5_ESM.docx]

**Supplementary Table 2** - Gene Ontology terms (Biological Process) related to genes similarly expressed in workers and queens (see Figure 5C)

| GO terms related to genes similarly expressed in workers and queens | |
| --- | --- |
| **BIOLOGICAL PROCESS** | **N^o^ genes > 20**  **P ≤ 0.05** |
| **Development/Morphogenesis/Metamorphosis/Differentiation** |  |
| GO:0009791~post-embryonic development | 111 |
| GO:0002165~instar larval or pupal development | 107 |
| GO:0048731~system development | 304 |
| GO:0031175~neuron projection development | 74 |
| GO:0009888~tissue development | 123 |
| GO:0042335~cuticle development | 20 |
| GO:0048468~cell development | 171 |
| GO:0048513~organ development | 247 |
| GO:0048736~appendage development | 60 |
| GO:0009887~organ morphogenesis | 141 |
| GO:0048729~tissue morphogenesis | 67 |
| GO:0009886~post-embryonic morphogenesis | 92 |
| GO:0000902~cell morphogenesis | 110 |
| GO:0032989~cellular component morphogenesis | 126 |
| GO:0032990~cell part morphogenesis | 78 |
| GO:0035107~appendage morphogenesis | 58 |
| GO:0048858~cell projection morphogenesis | 76 |
| GO:0010927~cellular component assembly involved in morphogenesis | 26 |
| GO:0007552~metamorphosis | 94 |
| GO:0001709~cell fate determination | 37 |
| GO:0045165~cell fate commitment | 59 |
| GO:0016337~cell-cell adhesion | 21 |
| GO:0003002~regionalization | 100 |
| GO:0007389~pattern specification process | 109 |
| GO:0007411~axon guidance | 34 |
| GO:0030154~cell differentiation | 218 |
| GO:0010259~multicellular organismal aging | 26 |
| GO:0048469~cell maturation | 33 |
|  | **2,722** |
| **Metabolic Process** |  |
| GO:0005975~carbohydrate metabolic process | 99 |
| GO:0044248~cellular catabolic process | 101 |
| GO:0044262~cellular carbohydrate metabolic process | 49 |
| GO:0016052~carbohydrate catabolic process | 26 |
| GO:0046483~heterocycle metabolic process | 67 |
| GO:0044255~cellular lipid metabolic process | 52 |
| GO:0005996~monosaccharide metabolic process | 29 |
| GO:0006082~organic acid metabolic process | 86 |
| GO:0006091~generation of precursor metabolites and energy | 77 |
| GO:0006139~nucleobase, nucleoside, nucleotide and nucleic acid metabolic process | 308 |
| GO:0006519~cellular amino acid and derivative metabolic process | 50 |
| GO:0006629~lipid metabolic process | 66 |
| GO:0006725~cellular aromatic compound metabolic process | 23 |
| GO:0006793~phosphorus metabolic process | 124 |
| GO:0009057~macromolecule catabolic process | 67 |
| GO:0009308~amine metabolic process | 79 |
| GO:0034641~cellular nitrogen compound metabolic process | 357 |
| GO:0042180~cellular ketone metabolic process | 93 |
| GO:0044260~cellular macromolecule metabolic process | 549 |
| GO:0051186~cofactor metabolic process | 47 |
|  | **2,349** |
| **Biosynthetic Process** |  |
| GO:0008610~lipid biosynthetic process | 35 |
| GO:0042254~ribosome biogenesis | 22 |
| GO:0009059~macromolecule biosynthetic process | 281 |
| GO:0044249~cellular biosynthetic process | 405 |
|  | **743** |
| **Cell Cycle** |  |
| GO:0022402~cell cycle process | 137 |
| GO:0022403~cell cycle phase | 125 |
| GO:0000278~mitotic cell cycle | 117 |
| GO:0051231~spindle elongation | 37 |
| GO:0000910~cytokinesis | 24 |
| GO:0007052~mitotic spindle organization | 73 |
|  | **513** |
| **Transport/Localization** |  |
| GO:0022900~electron transport chain | 34 |
| GO:0055085~transmembrane transport | 35 |
| GO:0051235~maintenance of location | 22 |
| GO:0008104~protein localization | 101 |
| GO:0070727~cellular macromolecule localization | 62 |
| GO:0016477~cell migration | 46 |
| GO:0046907~intracellular transport | 70 |
| GO:0048870~cell motility | 48 |
| GO:0006928~cell motion | 77 |
|  | **495** |
| **Regulation** |  |
| GO:0050793~regulation of developmental process | 77 |
| GO:0044087~regulation of cellular component biogenesis | 22 |
| GO:0051726~regulation of cell cycle | 47 |
| GO:0051128~regulation of cellular component organization | 69 |
| GO:0010941~regulation of cell death | 34 |
| GO:0010648~negative regulation of cell communication | 28 |
| GO:0048518~positive regulation of biological process | 81 |
| GO:0048522~positive regulation of cellular process | 73 |
|  | **431** |
| **Cellular Component Organization** |  |
| GO:0048285~organelle fission | 45 |
| GO:0051276~chromosome organization | 74 |
| GO:0000226~microtubule cytoskeleton organization | 98 |
| GO:0007010~cytoskeleton organization | 139 |
|  | **356** |
| **Gene expression** |  |
| GO:0010467~gene expression | **321** |
|  |  |
| **Macromolecular Complex Assembly/Organization** |  |
| GO:0034622~cellular macromolecular complex assembly | 53 |
| GO:0065003~macromolecular complex assembly | 68 |
| GO:0006461~protein complex assembly | 45 |
| GO:0034621~cellular macromolecular complex subunit organization | 61 |
|  | **227** |
| **Response to Stimulus** |  |
| GO:0006979~response to oxidative stress | 26 |
| GO:0033554~cellular response to stress | 44 |
|  | **70** |
| **Cell Death** |  |
| GO:0012501~programmed cell death | **44** |
