# Supplementary material for: Transcriptome dynamics during metamorphosis of imaginal discs into wings and thoracic dorsum in Apis mellifera castes
Source: BMC Genomics. 2021 Oct 22;22:756. doi: 10.1186/s12864-021-08040-z (PMC8532292; doi:10.1186/s12864-021-08040-z)
Supplement: Supplementary file 6 — Additional file 6. [file 12864_2021_8040_MOESM6_ESM.docx]

| GO terms related to genes upregulated in workers (W) or queens (Q) | | |
| --- | --- | --- |
| **BIOLOGICAL PROCESS** | **N^o^ genes ≥ 20 P ≤ 0.05** | |
| **Metabolic Process** | **Q** | **W** |
| GO:0044260~Cellular macromolecule metabolic process | 142 | 139 |
| GO:0034641~Cellular nitrogen compound metabolic process | 92 | 88 |
| GO:0006139~Nucleobase, nucleoside, nucleotide and nucleic acid metabolic process | 87 | 81 |
| GO:0006629~Lipid metabolic process | 20 |  |
| GO:0006519~Cellular amino acid and derivative metabolic process |  | 25 |
| GO:0009308~Amine metabolic process |  | 25 |
| GO:0042180~Cellular ketone metabolic process |  | 22 |
| GO:0006082~Organic acid metabolic process |  | 21 |
| GO:0009057~Macromolecule catabolic process |  | 20 |
|  | **341** | **421** |
| **Biosynthetic Process** |  |  |
| GO:0044249~Cellular biosynthetic process | 82 | 91 |
| GO:0009059~Macromolecule biosynthetic process | 61 | 69 |
|  | **143** | **160** |
| **Transport/Localization** |  |  |
| GO:0006810~Transport |  | 84 |
| GO:0016192~Vesicle mediated transport |  | 34 |
| GO:0008104~Protein localization | 25 | 29 |
| GO:0015031~Protein transport |  | 22 |
| GO:0045184~Establishment of protein localization |  | 22 |
| GO:0006897~Endocytosis |  | 21 |
| GO:0010324~Membrane invagination |  | 21 |
|  | **25** | **233** |
| **Regulation** |  |  |
| GO:0019222~Regulation of metabolic process | 66 |  |
| GO:0060255~Regulation of macromolecule metabolic process | 60 |  |
| GO:0031323~Regulation of cellular metabolic process | 58 |  |
| GO:0080090~Regulation of primary metabolic process | 55 |  |
| GO:0048518~Positive regulation of biological process | 22 |  |
|  | **261** |  |
| **Gene Expression** |  |  |
| GO:0010467~Gene expression | 80 | 83 |
|  | **80** | **83** |
| **Cell Differentiation** |  |  |
| GO:0030154~Cell differentiation | 57 |  |
|  | **57** |  |

**Supplementary Table 3** – Gene Ontology terms (Biological Process) related to genes upregulated in workers or queens (see Figure 5D).
