# Supplementary material for: Transcriptome dynamics during metamorphosis of imaginal discs into wings and thoracic dorsum in Apis mellifera castes
Source: BMC Genomics. 2021 Oct 22;22:756. doi: 10.1186/s12864-021-08040-z (PMC8532292; doi:10.1186/s12864-021-08040-z)
Supplement: Supplementary file 9 — Additional file 9. [file 12864_2021_8040_MOESM9_ESM.docx]

**SUPPLEMENTARY TABLE 6 -** Number of reads in wing disc ame-miRNAs libraries**.**

| **Number of Reads** | **Forewings** | **Hindwings** |
| --- | --- | --- |
| **Raw File** | 146430110 | 159890184 |
| **Processed files** | 91164927 | 126159328 |
| **Mapped** | 69283361 | 102533969 |
