# Supplementary material for: Transcriptome dynamics during metamorphosis of imaginal discs into wings and thoracic dorsum in Apis mellifera castes
Source: BMC Genomics. 2021 Oct 22;22:756. doi: 10.1186/s12864-021-08040-z (PMC8532292; doi:10.1186/s12864-021-08040-z)
Supplement: Supplementary file 10 — Additional file 10. [file 12864_2021_8040_MOESM10_ESM.docx]

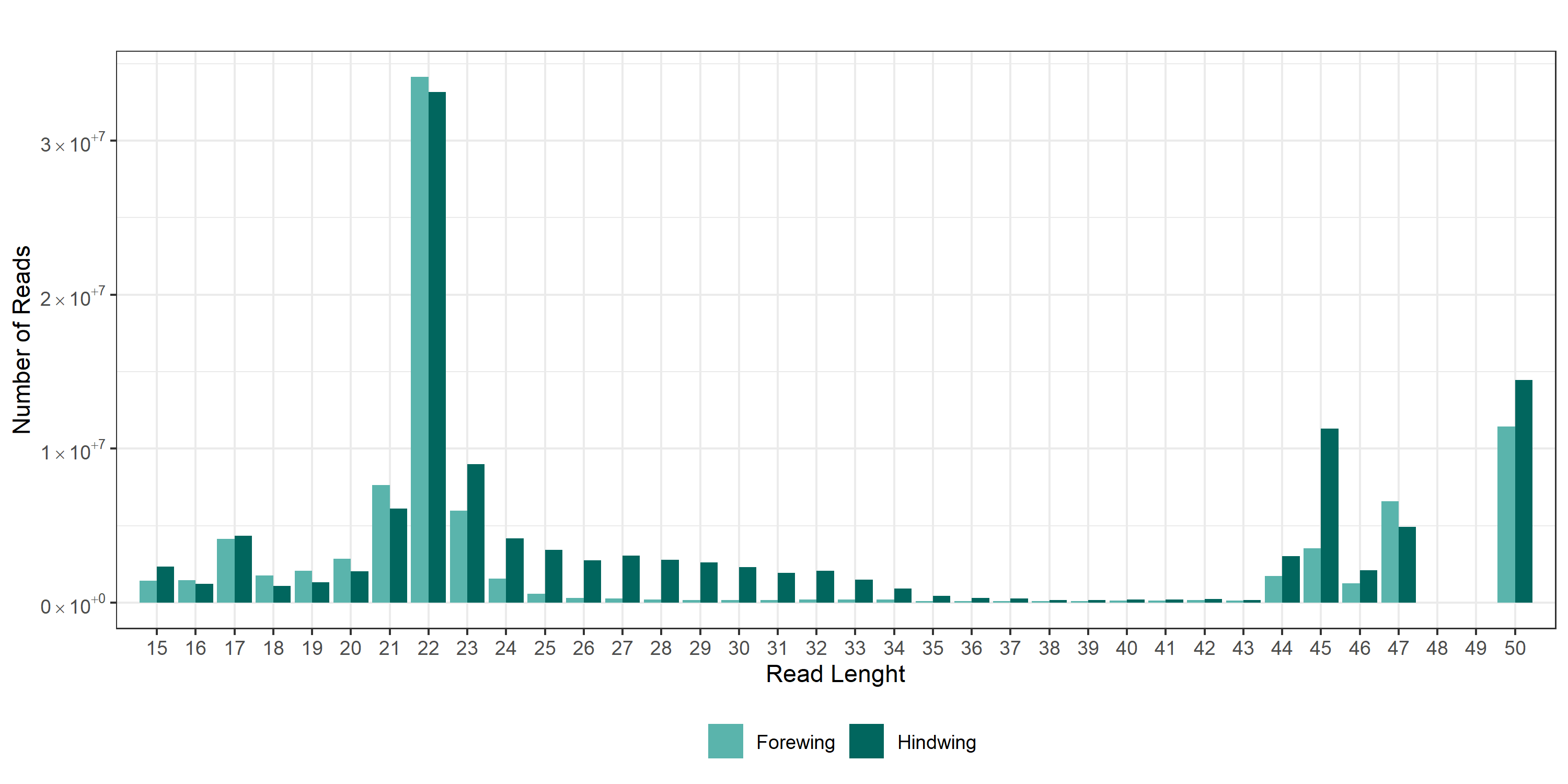
**SUPPLEMENTARY FIGURE 4** – Length distribution of miRNA reads after quality filtering. Reads in the range of 19-24 nt correspond to 60% and 44% of total reads from forewings and hindwings, respectively.
