# Supplementary material for: Transcriptome dynamics during metamorphosis of imaginal discs into wings and thoracic dorsum in Apis mellifera castes
Source: BMC Genomics. 2021 Oct 22;22:756. doi: 10.1186/s12864-021-08040-z (PMC8532292; doi:10.1186/s12864-021-08040-z)
Supplement: Supplementary file 11 — Additional file 11. [file 12864_2021_8040_MOESM11_ESM.docx]

**SUPPLEMENTARY TABLE 7 -** ame-miRNA number of reads in the honeybee worker wing discs in metamorphosis (L5PP2 phase)**.** The expression value is the sum of reads from both fore- and hindwing discs libraries.

| **ame-miRNAs** | **Number of reads** |
| --- | --- |
| *ame-miR-10-5p | 19842897 |
| *ame-miR-100-5p | 10619755 |
| *ame-miR-125-5p | 6930418 |
| *ame-miR-8-3p | 6333209 |
| *ame-miR-276-3p | 5503178 |
| *ame-bantam-3p | 4481247 |
| ame-miR-92b-1-3p | 3783896 |
| *ame-miR-306-5p | 3655897 |
| *ame-miR-2765-5p | 2950042 |
| ame-miR-92c-3p | 2461137 |
| *ame-miR-184-3p | 2090867 |
| ame-miR-279d-3p | 1686369 |
| *ame-let-7-5p | 1316429 |
| *ame-miR-275-3p | 1003770 |
| ame-miR-996-3p | 959069 |
| **ame-miR-2-1-3p | 897844 |
| ame-miR-2b-5p | 897844 |
| **ame-miR-2-3-3p | 856630 |
| **ame-miR-2-2-3p | 810566 |
| ame-miR-279a-3p | 730408 |
| ame-miR-31a-5p | 605198 |
| *ame-miR-13b-3p | 604371 |
| ame-miR-11-3p | 565977 |
| ame-miR-14-5p | 351496 |
| *ame-miR-305-5p | 348854 |
| *ame-miR-9a-3p | 336578 |
| *ame-miR-8-5p | 303984 |
| *ame-miR-14-3p | 235368 |
| *ame-miR-10-3p | 188410 |
| *ame-miR-13a-3p | 185341 |
| *ame-miR-263a-5p | 173280 |
| *ame-miR-315-5p | 137072 |
| *ame-miR-283-5p | 131239 |
| *ame-miR-9a-5p | 129084 |
| ame-miR-125-3p | 117513 |
| ame-miR-6037-3p | 117470 |
| ame-miR-278-5p | 104106 |
| ame-miR-3718b-5p | 90797 |
| ame-miR-3718a-3p | 90649 |
| ame-miR-3718c-3p | 90167 |
| ame-miR-3786-5p | 85589 |
| *ame-miR-9b-3p | 81780 |
| *ame-miR-193-3p | 78031 |
| ame-let-7-3p | 74737 |
| *ame-miR-92a-3p | 70666 |
| ame-miR-6001-3p | 65320 |
| ame-miR-927a-5p | 65113 |
| *ame-miR-993-3p | 64311 |
| *ame-miR-276-5p | 59291 |
| *ame-miR-2788-3p | 50089 |
| ame-miR-13b-5p | 49452 |
| *ame-miR-12-5p | 47458 |
| ame-miR-92a-5p | 42577 |
| *ame-miR-71-5p | 38298 |
| *ame-miR-317-3p | 34414 |
| ame-miR-279c-3p | 33734 |
| ame-miR-279c-5p | 26998 |
| ame-miR-3477-5p | 25071 |
| *ame-miR-190-5p | 25067 |
| ame-miR-317-5p | 19995 |
| ame-miR-3720-5p | 19968 |
| ame-miR-137-3p | 18620 |
| *ame-miR-263b-5p | 17407 |
| *ame-miR-316-5p | 16784 |
| ame-miR-29b-3p | 16237 |
| ame-miR-275-5p | 15480 |
| *ame-miR-965-3p | 15424 |
| ame-miR-993-5p | 15134 |
| *ame-miR-79-3p | 14828 |
| ame-miR-9b-5p | 14815 |
| *ame-miR-375-3p | 13294 |
| ame-miR-87-2-3p | 12585 |
| ame-miR-87-1-3p | 12374 |
| *ame-miR-277-3p | 12319 |
| ame-miR-1-1-3p | 11800 |
| ame-miR-1-2-3p | 11727 |
| ame-miR-79-5p | 11566 |
| ame-miR-3719-3p | 9767 |
| *ame-miR-2796-3p | 9494 |
| ame-miR-306-3p | 9392 |
| ame-miR-282-5p | 9130 |
| ame-miR-6051-3p | 9055 |
| ame-miR-3743-3p | 9034 |
| ame-miR-2788-5p | 8902 |
| ame-miR-252a-5p | 8645 |
| ame-miR-927a-3p | 7839 |
| ame-miR-100-3p | 7713 |
| ame-miR-3719-5p | 7193 |
| ame-miR-6005-5p | 7188 |
| *ame-miR-281-3p | 7138 |
| ame-miR-33-3p | 6587 |
| *ame-miR-281-5p | 6543 |
| ame-miR-2-2-5p | 6255 |
| ame-miR-996-5p | 6073 |
| ame-miR-3720-3p | 5702 |
| *ame-miR-133-3p | 5486 |
| ame-miR-315-3p | 4903 |
| ame-miR-92b-1-5p | 4735 |
| *ame-miR-307-3p | 4570 |
| ame-miR-7-5p | 4545 |
| ame-miR-2-3-5p | 4470 |
| ame-miR-87-2-5p | 4298 |
| ame-miR-6053-5p | 3765 |
| ame-miR-263a-3p | 3744 |
| ame-miR-9868-3p | 3744 |
| ame-miR-3785-5p | 3588 |
| ame-miR-3718a-5p | 3382 |
| ame-miR-3718b-3p | 3382 |
| ame-miR-6000a-5p | 3303 |
| ame-miR-6000b-3p | 3293 |
| ame-miR-6043-3p | 3122 |
| *ame-miR-34-5p | 2957 |
| ame-miR-11-5p | 2866 |
| ame-miR-3785-3p | 2484 |
| ame-miR-3715-5p | 2345 |
| ame-miR-6065-3p | 2312 |
| ame-miR-184-5p | 2162 |
| ame-miR-279d-5p | 1754 |
| ame-bantam-5p | 1642 |
| ame-miR-3478-5p | 1639 |
| ame-miR-3791-3p | 1639 |
| ame-miR-3718c-5p | 1626 |
| ame-miR-6005-3p | 1540 |
| ame-miR-3782-5p | 1456 |
| *ame-miR-278-3p | 1423 |
| ame-miR-3049-5p | 1282 |
| ame-miR-87-1-5p | 1167 |
| ame-miR-279b-3p | 1137 |
| ame-miR-6044-3p | 989 |
| ame-miR-1000-5p | 979 |
| ame-miR-3727-3p | 968 |
| ame-miR-3747b-5p | 956 |
| ame-miR-34-3p | 883 |
| ame-miR-9864-5p | 813 |
| ame-miR-989-3p | 801 |
| ame-miR-3786-3p | 728 |
| ame-miR-305-3p | 680 |
| ame-miR-6052-5p | 650 |
| ame-miR-9878-3p | 610 |
| ame-miR-3791-5p | 601 |
| ame-miR-3478-3p | 601 |
| ame-miR-2944-3p | 598 |
| ame-miR-965-5p | 594 |
| ame-miR-9884-3p | 584 |
| ame-miR-2944-5p | 568 |
| ame-miR-6001-5p | 550 |
| ame-miR-3049-3p | 516 |
| ame-miR-980-5p | 476 |
| ame-miR-3793-5p | 474 |
| ame-miR-6065-5p | 471 |
| ame-miR-9883-5p | 470 |
| ame-miR-932-5p | 446 |
| ame-miR-929-3p | 444 |
| ame-miR-92c-5p | 430 |
| ame-miR-283-3p | 423 |
| ame-miR-282-3p | 422 |
| ame-miR-9865-5p | 413 |
| ame-miR-6040-3p | 413 |
| ame-miR-7-3p | 392 |
| ame-miR-316-3p | 343 |
| ame-miR-3732-3p | 340 |
| ame-miR-279a-5p | 340 |
| ame-miR-6037-5p | 316 |
| ame-miR-13a-5p | 303 |
| ame-miR-9878-5p | 300 |
| ame-miR-3773-5p | 295 |
| ame-miR-980-3p | 290 |
| ame-miR-9892-3p | 283 |
| ame-miR-929-5p | 272 |
| ame-miR-252b-5p | 271 |
| ame-miR-6051-5p | 252 |
| ame-miR-981-3p | 239 |
| ame-miR-3756-3p | 234 |
| ame-miR-2765-3p | 233 |
| ame-miR-3477-3p | 210 |
| ame-miR-9886-3p | 205 |
| ame-miR-190-3p | 202 |
| ame-miR-6044-5p | 196 |
| ame-miR-927b-5p | 186 |
| ame-miR-6067-5p | 181 |
| ame-miR-9886-5p | 180 |
| ame-miR-9883-3p | 177 |
| ame-miR-1-2-5p | 174 |
| ame-miR-9872-5p | 173 |
| ame-miR-1-1-5p | 167 |
| ame-miR-9891-3p | 166 |
| ame-miR-9895-3p | 139 |
| ame-miR-971-3p | 137 |
| *ame-miR-3770-5p | 136 |
| ame-miR-971-5p | 135 |
| ame-miR-9888-3p | 122 |
| ame-miR-3728-5p | 121 |
| ame-miR-3756-5p | 119 |
| ame-miR-6042-3p | 115 |
| ame-miR-6061-3p | 104 |
| ame-miR-3730-5p | 102 |
| ame-miR-9871-3p | 100 |
