# Supplementary material for: Transcriptome dynamics during metamorphosis of imaginal discs into wings and thoracic dorsum in Apis mellifera castes
Source: BMC Genomics. 2021 Oct 22;22:756. doi: 10.1186/s12864-021-08040-z (PMC8532292; doi:10.1186/s12864-021-08040-z)
Supplement: Supplementary file 13 — Additional file 13. [file 12864_2021_8040_MOESM13_ESM.docx]

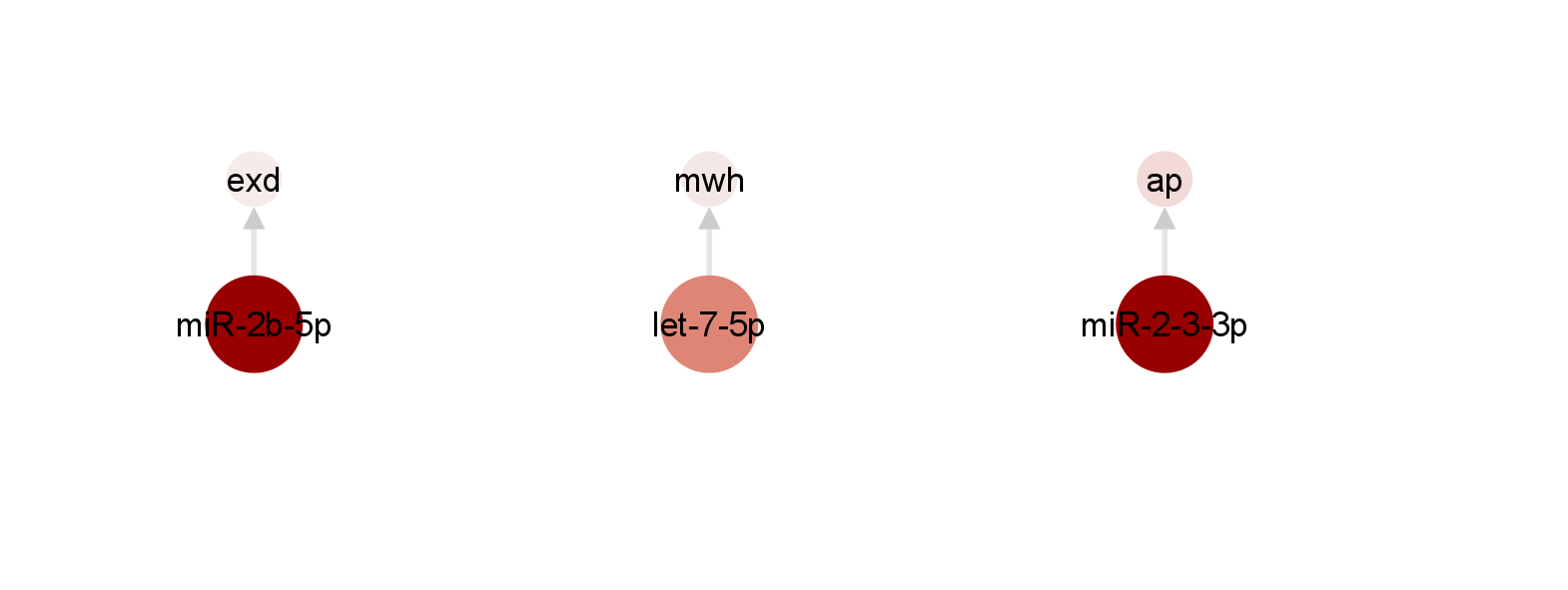


**SUPPLEMENTARY FIGURE 6** – Interaction between ame-miRNAs (miR-2b-5p, let-7-5p, miR-2-3-3p) and *A. mellifera* genes (*exd*, *mwh*, *ap*) displaying orthology relationships with wing disc patterning genes in *D. melanogaster*.
